# Longitudinal Pharmacometabonomics for Predicting Malignant Tumor Patient Responses to Anlotinib Therapy: Phenotype, Efficacy, and Toxicity

**DOI:** 10.3389/fonc.2020.548300

**Published:** 2020-11-12

**Authors:** Ting Hu, Zhuoling An, Yongkun Sun, Xunqiang Wang, Ping Du, Pengfei Li, Yihebali Chi, Lihong Liu

**Affiliations:** ^1^Pharmaceutical Department, Beijing Chao-Yang Hospital, Capital Medical University, Beijing, China; ^2^National Cancer Center, National Clinical Research Center for Cancer, Cancer Hospital, Chinese Academy of Medical Sciences and Peking Union Medical College, Beijing, China; ^3^Research and Development Department, Chia Tai Tianqing Pharmaceutical Group Co., Nanjing, China

**Keywords:** pharmacometabonomics, anlotinib, phenotype, efficacy, toxicity, ultra-high performance liquid chromatography-tandem mass spectrometry

## Abstract

Anlotinib is an oral small molecule inhibitor of multiple receptor tyrosine kinases (RTKs), which was approved by the National Medical Products Administration (NMPA) of China in 2018 for the third-line treatment of non-small cell lung cancer (NSCLC). Here, for the first time, the longitudinal pharmacometabonomics was explored for predicting malignant tumor patient responses to anlotinib, including the metabolic phenotype variation, drug efficacy, and toxicity. A total of 393 plasma samples from 16 subjects collected from a phase I additional study of anlotinib (NCT02752516) were submitted to targeted metabolomics analysis. The orthogonal partial least-squares discriminant analysis (OPLS-DA) models were constructed for the predication of anlotinib efficacy and toxicity based on the longitudinal pharmacometabonomics data. Statistical results showed that 38 metabolites, mainly involved in aminoacyl-tRNA biosynthesis, alanine, aspartate, and glutamate metabolism, and steroid hormone biosynthesis, were all significantly upregulated attributing to anlotinib treatment. The anti-tumor efficacy and occurrence of proteinuria after anlotinib administration can be predicted with 100% accuracy using the established OPLS-DA models. Glycodeoxycholic acid and glycocholic acid possessed the most excellent sensitivity and specificity in predicting the efficacy of anlotinib, with area under the receiver operating characteristic curve (AUC of ROC curve) 0.847 and 0.828, respectively. NG, NG-dimethylarginine was the most promising biomarker for the prediction of proteinuria occurrence after anlotinib administration, with AUC of ROC curve 0.814. In conclusion, this work developed efficient and convenient discriminant models that can accurately predict the efficacy and toxicity of anlotinib based on longitudinal pharmacometabonomics study.

## Introduction

Anlotinib is an oral small molecule inhibitor of multiple receptor tyrosine kinases (RTKs) developed by Jiangsu Chia-Tai Tianqing Pharmaceutical and Advenchen Laboratories for the treatment of advanced cancer (1). It was designed to primarily inhibit vascular endothelial growth factor receptors (VEGFRs) 2 and 3, fibroblast growth factor receptors (FGFRs) 1–4, platelet-derived growth factor receptors (PDGFRs) *α* and *β*, c-Kit and Ret, which brought it a broad spectrum of inhibitory effects on tumor angiogenesis and growth (1–3). It was approved by the National Medical Products Administration (NMPA) of China in 2018 for the third-line treatment of non-small cell lung cancer (NSCLC) (1). Anlotinib is also undergoing clinical trials for the treatment of various sarcomas and carcinomas in China, USA, and Italy (1, 4–6). The development of anlotinib is a major breakthrough in the Chinese history of anti-tumor drugs.

The pharmacokinetic characteristics of anlotinib have been elucidated by the first-in-human, open-label phase I study (NCT01833923) in subjects with advanced refractory solid tumors (7). The time for the blood concentration to reach the peak was 7.3 h after a single dose of 12 mg anlotinib. The elimination of anlotinib was very slow, with an elimination half-life of 98 h. The long half-life resulted in marked accumulation of the drug over time. Anlotinib 12 mg once-daily on the first 14 days of each 21-day cycle showed promising anti-tumor activity. This 2-week on/1-week off administration protocol was defined as one treatment cycle and ultimately used in the clinical practice. In the phase I study (NCT01833923), all the subjects experienced adverse events (AEs). The most common grade 3 AEs related to anlotinib were proteinuria, hypertension, fatigue, dyslipidemia, and hand–foot skin reactions (2, 8, 9). Though, it was revealed that anlotinib administration showed less and milder diarrhea than other oral anti-VEGFR receptor tyrosine kinase inhibitors (10–12). It should pay attention that patients undergoing anlotinib treatment still had a high occurrence of AEs. At present, regular monitoring of patients was the only way to detect AEs caused by anlotinib (2, 13). More accurate and specific method needs to be developed for the early prediction of AEs caused by anlotinib treatment.

Personalized medicine is the choice of medicines for subgroups or even individual patient, aiming to maximize drug efficacy and minimize toxicity. It is a key goal of the 21^st^ century healthcare (14, 15). Pharmacometabonomics is the profiling of metabolite levels in biofluids or tissues to predict the benefits and toxicity of a drug intervention (14, 15). It has emerged as an important tool of personalized medicine in the past decade, applying for discovering the therapeutic mechanism, predicting efficacy and AEs of a drug (14). As the patients’ responses to drugs are influenced by both genetic and environmental elements, the pharmacometabonomics plays an increasing important role in personalized medicine because of its sensitivity to both genes and environment (15). It has been successfully used for the prediction of drug metabolism, efficacy, and toxicity (16–20). Recent applications of pharmacometabonomics have extended to the use of longitudinal sampling from clinical trials of new drugs (21). Unlike the traditional pharmacometabonomics study, which was the use of pre-dose metabolomics data to predict drug disposition, the longitudinal pharmacometabonomics conducted metabolomics profiles prior to, during, and after drug intervention to stratify the patient in terms of prediction of response to future treatment (21, 22).

In this study, the utility of longitudinal pharmacometabonomics was explored for predicting malignant tumor patient responses to anlotinib, including the metabolic phenotype variation, drug efficacy and toxicity. Plasma samples were collected from a phase I additional study of anlotinib (NCT02752516) (23). A robust and single-injection targeted metabolomics profiling method was used for metabolites quantification in the plasma samples by ultra-high performance liquid chromatography-tandem mass spectrometry (UHPLC-MS/MS). Finally, 181 metabolites were successfully quantified from the plasma and their concentrations were further applied for statistical analysis. Metabolic phenotype and pathway variation related to anlotinib treatment were explored. The orthogonal partial least-squares discriminant analysis (OPLS-DA) models were established for the predication of efficacy and toxicity of anlotinib based on the longitudinal pharmacometabonomics data. The OPLS-DA models were further evaluated by both internal and external validation.

## Materials and Methods

### Chemicals and Reagents

Unlabeled metabolite standards (**Supplementary Table S1**) were purchased from Sigma-Aldrich (St. Louis, MO, USA), Cayman Chemical (Ann Arbor, MI, USA), Bidepharm (Shanghai, China) or Steraloids (Newport, RI, USA). Stable isotope-labeled internal standards (ISs) were purchased from Cambridge Isotope Laboratories (Cambridge, MA, USA). Acetonitrile (MS grade) and isopropyl alcohol (HPLC grade) were purchased from Fisher Scientific (Pittsburgh, PA, USA). Formic acid (HPLC grade) was obtained from TEDIA Co., Inc. (Fairfield, OH, USA). A Milli-Q purification system (Bedford, MA, USA) was used for the preparation of ultrapure water.

### Study Population

All the plasma samples employed in this longitudinal pharmacometabonomics study were collected from a phase I additional study of anlotinib. This phase I clinical trial of anlotinib was conducted in the Cancer Hospital, Chinese Academy of Medical Sciences and Peking Union Medical College. The clinical trial was approved by the Chinese government with a clinical trial registration number CTR20160544. It was also registered on the ClinicalTrials.gov with registration number NCT02752516. Detail information of this clinical trial of anlotinib can be found on the internet (http://www.chinadrugtrials.org.cn/ and https://clinicaltrials.gov/) by entering the clinical trial registration number. Dosage selection for this clinical trial was based on preclinical pharmacological and toxicological experimental results. Patients with pathologically and/or cytologically proven advanced cancer with no standard therapy were included in the clinical trial. Eligibility criteria included ages 18–65, ECOG PS 0–1, and an estimated survival duration of more than 3 months. Patients who had used other chemotherapy drugs needed to stop for at least 30 days; patients who had received major surgery needed to rest for at least 4 weeks. The dosage selection, inclusion and exclusion criteria of this clinical trial were described in detail in a previous report (7).

**Figure 1:**
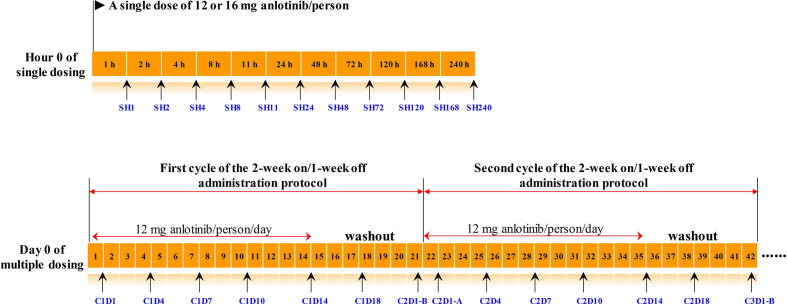
The timeline of drug administration and blood collection. Numbers in the orange boxes represented the time. The black arrows below the timeline indicated the points at which the blood samples were collected, and the blue texts indicated the corresponding sample number. The upper panel was timeline of the single-dose study, and the lower panel was timeline of the multiple-dose study. C2D1-B and C2D1-A represented the time points 24 h before and 24 h after the first administration in the second cycle, respectively.

393 plasma samples from 16 subjects in this phase I study were applied to longitudinal pharmacometabonomics study. A single dose of 12 or 16 mg anlotinib/person was orally administered to all the 16 subjects in the single-dose study. Serial blood samples were collected in heparinized tubes at 1, 2, 4, 8, 11, 24, 48, 72, 120, 168, and 240 h after dosing. Ten days after the single dosing, all the 16 volunteers were given multiple doses of anlotinib at 12 mg anlotinib/person/day for two cycles, with twenty-one days for each treatment cycle. Blood samples were collected on days 1, 4, 7, 10, 14, 18, 21, 22, 25, 28, 31, 35, 38, and 42 in the multiple-dose study. A summary of the time points of drug administration and blood collection program was shown in **Figure 1**. The subject numbered 008 withdrew from the trial on day 22 of multiple-dose study due to serious adverse events (SAEs). The remaining 15 subjects completed the entire clinical trial. If the subjects benefited from the trial, they would continue to use anlotinib after the clinical trial with the 2-week on/1-week off administration protocol and were followed up. Efficacies were evaluated according to NCI-proposed Response Evaluation Criteria in Solid Tumors (RECIST 1.1) every two cycles of the administration protocol. AEs were graded into 0–5 according to the National Cancer Institute Common Terminology Criteria for Adverse Events (NCI-CTCAE 4.0). Subjects with unresolved AEs at the end of the trial needed to be treated and followed until the reactions returned to the grade 1 degree or less, or stable.

This study was conducted in accordance with the Declaration of Helsinki, International Conference on Harmonization (ICH) and Good Clinical Practice guidelines. All study documentations, such as study protocol and consent forms, were reviewed and approved by the Institutional Review Board of Cancer Hospital before the initiation of the clinical trial. The longitudinal pharmacometabonomics study was conducted in Beijing Chao-Yang Hospital affiliated with Capital Medical University. As the plasma samples used in this pharmacometabonomics study were the remaining samples from the pharmacokinetic study, the longitudinal pharmacometabonomics study met the conditions for exemption from informed consent and was approved by the ethics committee of Beijing Chao-Yang Hospital (ethical approval number: 2019-Research-144).

### Sample Pretreatment

IS mixture solution was prepared by mixing eight isotopic IS, with final concentration of 400 ng ml^−1^ for each. Standard mixture solution was prepared from all the standards listed in **Supplementary Table S1**. The working solution was stepwise diluted with methanol to prepare standard curve with concentrations of 0.2, 0.5, 2, 5, 20, 50, 100, 200, 500, 1,000, 2,000, and 5,000 ng ml^−1^. Finally, each 50 μl standard mixture was successively spiked with 50 μl water, 10 μl IS mixture, and 90 μl methanol to get the standard curve samples.

Plasma samples were pretreated using protein precipitation method as previously reported in the literature (24). Simply, each 50 μl plasma was mixed with 10 μl IS mixture and 140 μl methanol. The resulting mixture was then vortex-mixed for 2 min and centrifuged at 13,800 g for 10 min at 4°C. The supernatants were collected for UHPLC-MS/MS analysis. Quality control (QC) samples mixed by equal aliquot of plasma from all the tested samples were processed as real samples and inserted into the analysis sequence to check the stability of sample pretreatment procedure and instrumental system.

### Instrumental Analysis

A Spark Holland liquid chromatography system (Spark, Holland) coupled with an API 5500 mass spectrometer (AB Sciex, Canada) with a Turbo Ionspray electrospray ionization (ESI) source was used for targeted metabolomics analysis. Metabolomics analysis was conducted according to the previously reported method (24). A HSS T3 (150 mm × 2.1 mm, 3.5 μm) was selected for chromatographic separation. The column temperature was maintained at 20°C, and the injection volume was 5 μl. Solvent A was water containing 0.1% formic acid. Solvent B was acetonitrile/isopropyl alcohol in a 7:2 (v/v) ratio. The flow rate was 0.5 ml min^−1^. The elution gradient was set as follows: 0–4 min, 1–10% B; 4–8 min, 10–50% B; 8–15 min, 50–80% B; 15–25 min, 80–100% B; 25–27 min, 100–100% B. The elution condition was returned to the initial state over a period of 2 min.

All the metabolites were analyzed in a single-injection using both negative and positive modes with scheduled multiple reaction monitoring (s-MRM). Electrospray voltage was −4,500 V for negative scan and 5,500 V for positive scan. Source temperature was 600°C. GS1 was 60 psi. GS2 was 60 psi. Curtain gas was 40 psi. The MRM parameters of metabolites were listed in **Supplementary Table S1**.

### Data Processing and Statistical Analysis

Raw data files of UHPLC-MS/MS analysis were processed using MultiQuant software (version 3.0.2, AB SCIEX, Canada). Concentrations of the metabolites with commercial standards were calculated by the least-squares method with a *1/x^2^* weighting factor. For some acyl carnitines and fatty acids without standards, their relative concentrations were calculated according to the peak area ratio of an analyte to the corresponding IS. SIMCA 14.1 (Umetrics AB, Umeå, Sweden) was employed for principle component analysis (PCA) and OPLS-DA analysis. IBM SPSS 21 (Armonk, New York, United States) was employed for t-test, and P < 0.05 was set as the level of statistical significance. Box plot analysis and receiver operating characteristic curve (ROC) analysis were conducted in an open source tool of MetaboAnalyst 4.0 (HYPERLINK: https://www.metaboanalyst.ca/).

## Results

### Overview of the Targeted Metabolomics Method and Data Quality

**Figure 2:**
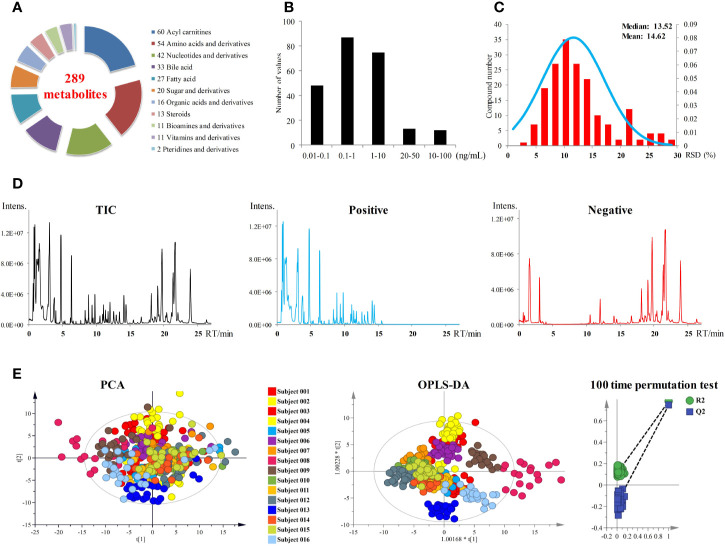
**(A)** Metabolites coverage of the targeted metabolomics method. **(B)** LOQs of the metabolomics method. **(C)** Performance of pooled plasma QC samples during sequence analysis. **(D)** Total ion currents (TICs) of plasma samples. **(E)** PCA and OPLS-DA score plots of 393 plasma samples from 16 subjects. OPLS-DA model was validated by random permutation test with 100 iterations. No overfitting was observed.

A targeted metabolomics method established in our laboratory was used for metabolites quantification in plasma samples (24). As shown in **Figure 2A**, a total of 289 metabolites of biological activity were covered in this targeted metabolomics method. All these 289 metabolites can be quantified in a single-injection of 27 min using both negative and positive modes with rapid polarity switching. The limits of quantitation (LOQs) ranged from 0.02 to 100 ng ml^−1^ (**Figure 2B**), depending on the chemical structures. Over 90% of the metabolites had LOQs lower than or equal to 10 ng ml^−1^, which guaranteed the successful detection of low abundance compounds. Other analytical parameters, including stability, linearity, and dynamic range were all carefully investigated (**Supplementary Table S1**). All these parameters can meet the requirement of accurate quantification.

All the 393 plasma samples harvested from the anlotinib phase I study (NCT02752516) were applied for metabolomics analysis. Finally, a total of 181 metabolites were detected and quantified in the plasma samples. The chromatograms of plasma sample were shown in **Figure 2D**. Pooled plasma QC samples were evenly interspersed throughout the sample analysis procedure to monitor the deviation introduced from sample pretreatment and instrumental analysis. The relative standard deviation (RSD) values of the QC samples were shown in **Figure 2C**. RSD values of all metabolites in QC samples were less than 30%, with more than 85% of them less than 20%. The good performance of pooled QC samples indicated that the metabolomics data achieved in this study was reliable and can be further used for statistical analysis.

### Longitudinal Metabolic Fingerprints of the Subjects

In order to explore the differences of metabolic fingerprints of the subjects at different time points, metabolite concentrations of the 393 plasma samples of all time points from 16 subjects were introduced to statistical analysis. Both PCA and OPLS-DA were applied to integrate and coanalyze all observations from plasma samples to investigate the longitudinal metabolic fingerprints of the subjects. The score plots of PCA and OPLS-DA were shown in **Figure 2E**. Individuals were used as the grouping basis and dots of the same color represented samples of one subject at different time points. As shown in the figure, plasma samples of the same subject segregated into tight cluster in both unsupervised PCA and supervised OPLS-DA, which indicated the longitudinal metabolic fingerprint of the same subject was relatively stable in different time points of anlotinib treatment. The metabolic changes caused by individual differences of subjects were greater than the metabolic disturbance caused by anlotinib treatment.

Most of the samples from subject 008 were outside the 95% confidence interval (CI) of both PCA and OPLS-DA score plots (**Figure 2E**). These indicated the significant differences of metabolic fingerprints between subject 008 and the other subjects, which were supposed to be correlated to the adverse clinical outcome. Subject 008 withdrew from the trial on day 22 in multiple-dose study due to SAEs of anemia, fatigue, and urinary infection. This subject later died of tumor progression.

### Metabolic Phenotype Variation Related to Anlotinib Treatment

According to the pharmacokinetic results of single-dose administration, anlotinib reached its maximum plasma concentration in 4–11 h after dosing, then it was eliminated slowly with mean residence time (MRT) ranged from 124 to 167 h (7). The multiple-dose study using 2-week on/1-week off administration protocol brought continuously increased plasma concentration of anlotinib in subjects in the first 2 weeks and the maximum plasma concentration occurring on day 14 (C1D14 and C2D14) of the twenty-one days treatment cycle. Subsequently, the plasma concentration of anlotinib apparently decreased with a 7-day washout until the beginning of the next 2-week on/1-week off treatment cycle (7). Blood concentrations (ng ml^−1^) of anlotinib at different time points were shown in **Table S2**. In order to explore the metabolic phenotype variation induced by anlotinib treatment, the metabolomics data of plasma sample at SH1 (1 h after the first anlotinib dosing) was compared with the metabolomics data at C1D14 and C2D14 (with maximum drug concentration). The time points C1D14 and C2D14 possessed the maximum plasma concentration of anlotinib (**Table S2**, **Figure S1**), which corresponded to the 14^th^ day of the first and second treatment cycles, respectively (**Figure 1**).

**Figure 3:**
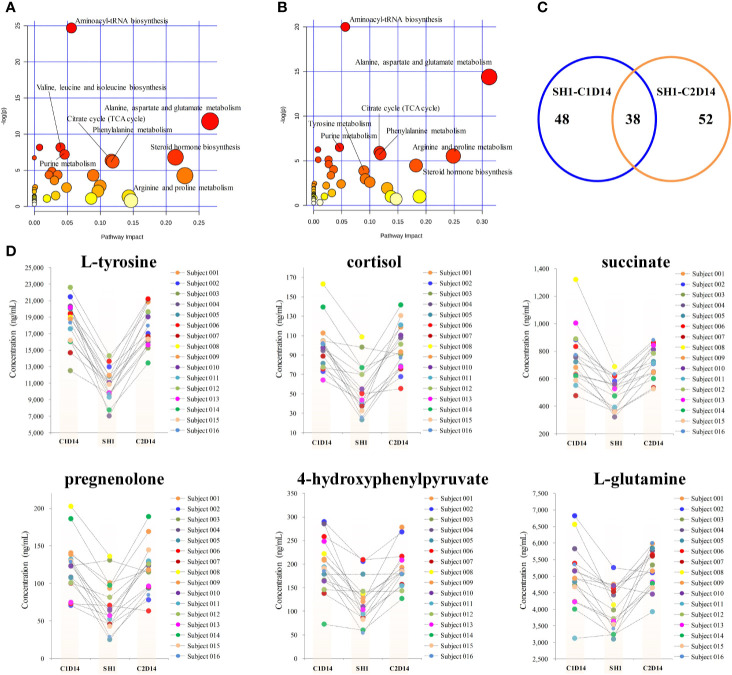
Metabolic phenotype variations induced by anlotinib treatment. **(A)** Overview of pathway analysis of the significantly changed metabolites between SH1 and C1D14. **(B)** Overview of pathway analysis of the significantly changed metabolites between SH1 and C2D14. All dots represented matched pathways from topology pathway analysis. Pathways were colored according to their significance values from pathway enrichment analysis, with gradations from yellow, having the least significance, to red having the highest significance. **(C)** A Venn diagram showing the shared metabolite numbers between SH1-C1D14 and SH1-C2D14. **(D)** Concentration trends of six metabolites on the time points of SH1, C1D14, and C2D14.

Paired t-test was first conducted to capture the most significantly changed metabolites between SH1 and C1D14, with all the 16 subjects included. A total of 48 metabolites were found to be significantly changed (P < 0.05) between SH1 and C1D14 (**Supplementary Table S3**). All the significantly changed metabolites were subjected to MetaboAnalyst 4.0 for pathway analysis, with result shown in **Figure 3A**. Fifteen metabolic pathways were obviously disturbed, with their pathway names, P values, and pathway impact factors listed in **Supplementary Table S4**.

**Figure 4:**
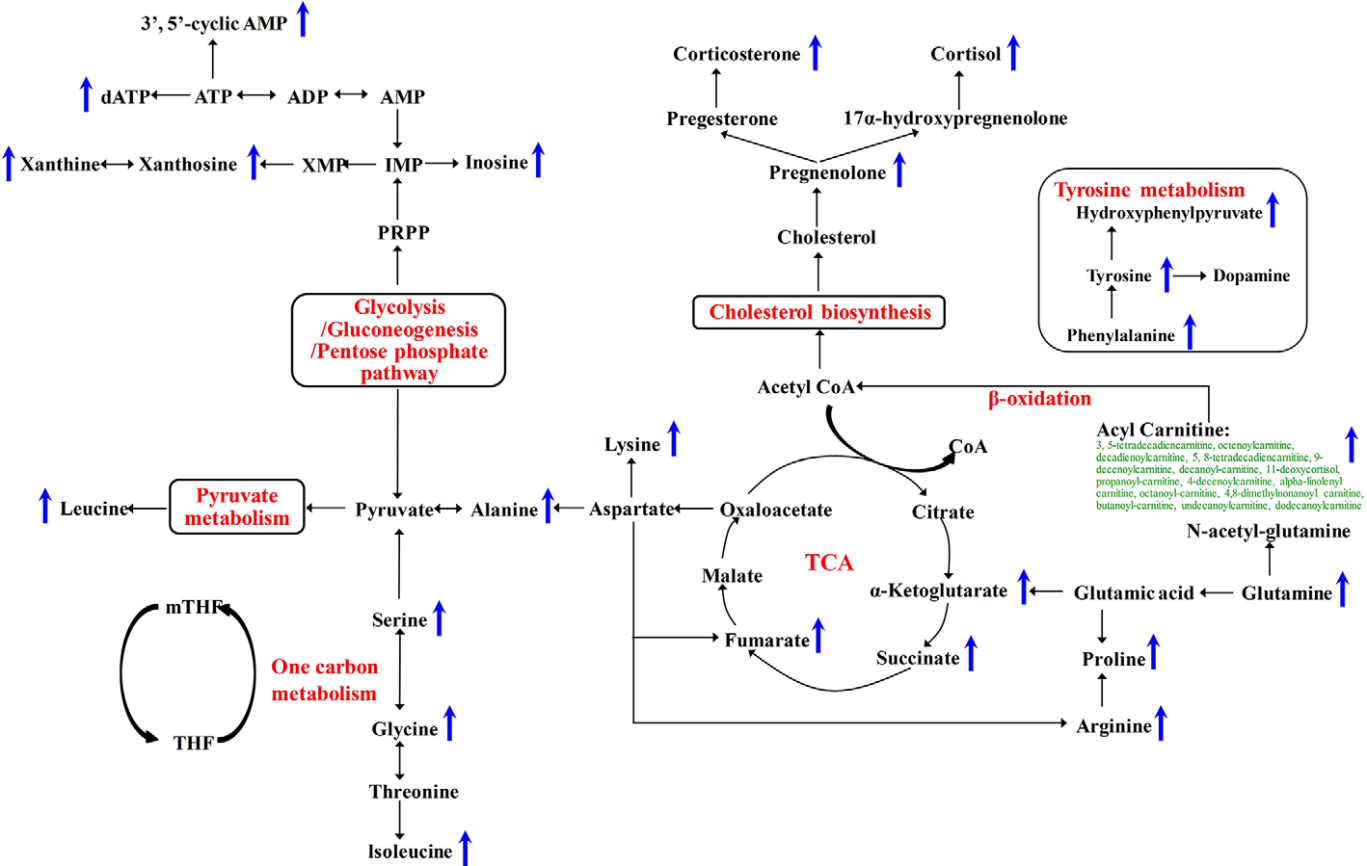
Metabolic pathways significantly disturbed by anlotinib treatment. The blue arrows indicated a significant (P < 0.05) upregulation of metabolite concentrations in plasma after treatment with anlotinib.

To further validate whether the significantly changed metabolic phenotypes between SH1 and C1D14 were caused by anlotinib treatment, the metabolic phenotype of C2D14 was also compared with the SH1 by paired t-test. A total of 52 metabolites were found to be significantly changed between SH1 and C2D14 (**Supplementary Table S5**). Pathway analysis was also conducted using these 52 metabolites, with result shown in **Figure 3B**. A total of 14 metabolic pathways were obviously disturbed, with their pathway names, P values, and pathway impact factors listed in **Supplementary Table S4**. Of the 48 significantly changed metabolites of SH1-C1D14 and 52 significantly changed metabolites of SH1-C2D14, 38 metabolites were shared by both (**Table S6**). A Venn diagram showing the numbers of the significantly changed metabolites was presented in **Figure 3C**. Of all the significantly disturbed metabolic pathways, fourteen were shared by both SH1-C1D14 and SH1-C2D14 (**Table S4**). The statistical results of SH1-C1D14 and SH1-C2D14 possessed a high degree of similarity. Inherent metabolic phenotype variations had taken place owing to the treatment of anlotinib. L-tyrosine, cortisol, succinate, pregnenolone, 4-hydroxyphenylpyruvate, and L-glutamine were the mostly significantly changed metabolites (**Table S6**). The concentration trends of these six metabolites on the time points of SH1, C1D14, and C2D14 were shown in **Figure 3D**. All these six metabolites possessed lower concentrations at the beginning of anlotinib administration (SH1) and further increased to a higher level after achieving the maximum plasma anlotinib concentration (C1D14 and C2D14). A typical concentration trend of L-tyrosine across all the time points was shown in **Supplementary Figure S2**. Although L-tyrosine can be affected by exogenous foods, the concentrations of L-tyrosine still exhibited an obvious correlation with the anlotinib administration cycle. L-tyrosine achieved its highest concentration at the time points of C_max_. A summary of the metabolic pathway disturbances related to anlotinib treatment was shown in **Figure 4**. All the metabolic pathways were significantly upregulated by anlotinib treatment.

### Identification of Potential Biomarkers Associated With the Efficacy of Anlotinib

According to the RECIST 1.1 criteria, 13 of the 16 subjects had target lesions and two of 16 had non-target lesions due to bone metastases. Subject 008 withdrew from the trial because of SAEs. Therefore, only 13 subjects were able to obtain the final objective remission rate data. The best curative effect is an important evaluation index of objective remission rate of tumor. It can be calculated by the formula:

$$Best\;curative\;effect=\frac{(\text{minimum}\;\text{value}\;\text{of}\;\text{tumor}\;\text{volume}-\text{baseline}\;\text{value}\;\text{of}\;\text{tumor}\;\text{volume})\times 100\%}{\text{baseline}\;\text{value}\;\text{of}\;\text{tumor}\;\text{volume}}.$$

The best curative effects of the 13 subjects were shown in **Supplementary Table S7**. All these 13 subjects were divided into a good efficacy group (*n* = 6) and a poor efficacy group (*n* = 7) according to their best curative effects. The good efficacy group achieved a best curative effect data better than −10%, while the poor efficacy group achieved a best curative effect data poor than −10%. A summary of the clinical characteristics and pharmacokinetic parameters of the 13 subjects was listed in **Table 1**. Both the baseline clinical characteristics and pharmacokinetic parameters showed no significant correlations with the efficacy of anlotinib.

**Table 1:** Clinical characteristics and pharmacokinetic parameters of the patients in the efficacy study by longitudinal pharmacometabonomics.

Characteristic	Good efficacy (*n* = 6)	Poor efficacy (*n* = 7)	P value*
Age (years)	45.5 ± 12.9	54.1 ± 14.8	0.289
BMI (kg/m^2^)	22.8 ± 3.1	24.0 ± 3.3	0.485
Gender			0.066
*Male*	3	4	
*Female*	3	3	
AUC_(0–t)_ (μg/L*h)	801.2 ± 179.3	724.9 ± 216.0	0.507
AUC_(0–∞)_ (μg/L*h)	1076.0 ± 196.8	933 ± 248.2	0.281
C_max_ (μg/L)	9.03 ± 2.28	7.36 ± 3.11	0.299
Tumor types			0.428
*Endometrial stromal tumor*	*n* = 1	*n* = 0	
*Adenoid cystic tumor*	*n* = 2	*n* = 2	
*Schwannoma*	*n* = 3	*n* = 1	
*Thyroid follicular tumor*	*n* = 0	*n* = 1	
*Hepatic carcinoma*	*n* = 0	*n* = 1	
*Corticosuprarenaloma*	*n* = 0	*n* = 1	
*Thymic carcinoma*	*n* = 0	*n* = 1	

*P values were calculated using bilateral t-test for age, BMI, AUC_(0–t)_, AUC_(0–∞)_ and C_max_ and using Chi^2^ test for gender and tumor types.

**Figure 5:**
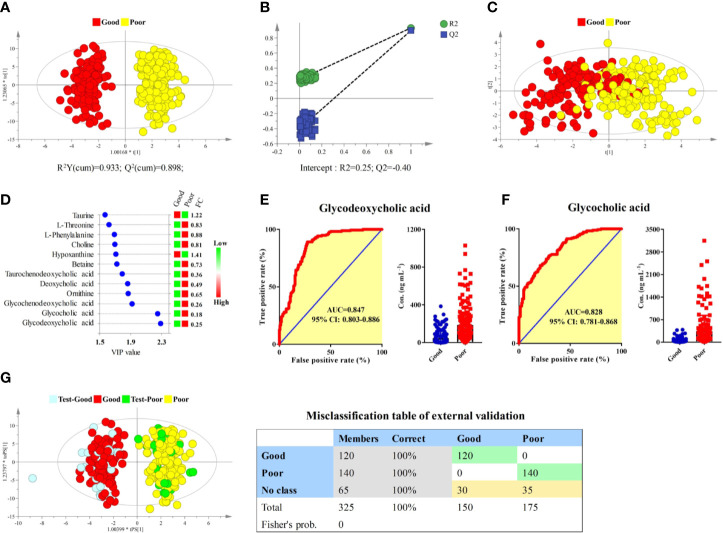
Identification of potential biomarkers associated with the efficacy of anlotinib. **(A)** OPLS-DA score plot of good and poor efficacy groups based on concentrations of all metabolites. **(B)** Random permutation test with 100 iterations. **(C)** PCA score plot of good and poor efficacy groups based on concentrations of the 12 metabolites with VIP > 1.5. **(D)** Compound names, VIP values, and FCs of the 12 metabolites with VIP > 1.5. **(E)** ROC and box plot of glycodeoxycholic acid. **(F)** ROC and box plot of glycocholic acid. **(G)** External validation of the OPLS-DA model. Samples in the discovery set were designed as good or poor groups, while samples in the validation set were designed as test-good or test-poor groups. Both depended on the best curative effect.

Longitudinal pharmacometabonomics data was employed for the identification of potential biomarkers associated with the efficacy of anlotinib. Due to the limited number of the subjects included in this study and the relatively stable longitudinal metabolic fingerprint of the same subject in different time points of anlotinib treatment, metabolomics data from all the 25 time points was included in the final statistical analysis to increase the reliability of the statistical results. First, the OPLS-DA and bilateral t-test were applied to integrate and coanalyze all observations from plasma samples to investigate the differences in metabolic phenotypes. The OPLS-DA score plot was shown in **Figure 5A**. The good efficacy group was completely separated from the poor efficacy group based on the concentration of the 181 metabolites. A random permutation test with 100 iterations was performed to validate the OPLS-DA model with result shown in **Figure 5B**. No overfitting was observed. The statistical result indicated that significant different metabolic phenotypes existed between good efficacy and poor efficacy groups.

To screen potential biomarkers associated with the efficacy of anlotinib, the variable importance in the projection (VIP) of each metabolite was calculated based on the established OPLS-DA model. Unsupervised PCA was conducted based on concentrations of 12 metabolites that possessed VIP values larger than 1.5, with score plot shown in **Figure 5C**. A moderate separation of the good and poor efficacy groups can be observed even on this unsupervised statistical method. Further statistical analysis showed that all the 12 metabolites possessed P values less than 1 × 10^−9^ and area under the curves (AUCs) of ROCs larger than 0.7. The VIP values and fold changes (FCs) of the 12 metabolites were shown in **Figure 5D**. More detailed information of the 12 metabolites was listed in **Supplementary Table S8**. Glycodeoxycholic acid and glycocholic acid were two metabolites with the most significant FCs and the largest AUCs of ROC analysis, with their ROCs and box plots shown in **Figures 5E, F**, respectively. Both of them belong to the glycine conjugated bile acids. The AUCs of glycodeoxycholic acid and glycocholic acid were 0.847 (95% CI: 0.803–0.886) and 0.828 (95% CI: 0.781–0.868), respectively. They were the most promising biomarkers related to efficacy of anlotinib. Lower plasma concentrations of glycodeoxycholic acid and glycocholic acid indicated better efficacy of anlotinib.

In order to carry out external validation of the OPLS-DA model, 80% of the total sample size was designed as the discovery set, and the other 20% was designed as the validation set. Samples in the discovery set were designed as good or poor groups depending on their best curative effects. As shown in **Figure 5G**, excellent OPLS-DA model was established based on samples from the discovery set. The established OPLS-DA model was further used for the discrimination of samples in the validation set. As shown in the figure, all the samples in the validation set can be distinguished with 100% accuracy based on the established OPLS-DA model.

### Exploring the Predictive Biomarkers Associated With the Toxicity of Anlotinib

According to the result of the clinical trial study, proteinuria was the most common AE related to anlotinib treatment, with more than 60% incidence. Here, potential biomarkers for the prediction of proteinuria occurrence were explored based on the longitudinal pharmacometabonomics data. Eight of the 16 subjects had experienced an AE of proteinuria, which were judged to be possibly or definitely related to the administration of anlotinib by research doctor. For biomarkers exploring, all these 16 subjects were divided into a proteinuria group (*n* = 8) and a no proteinuria group (*n* = 8). A summary of the clinical characteristics and pharmacokinetic parameters of the 16 subjects was listed in **Table 2**. Both the baseline clinical characteristics and pharmacokinetic parameters showed no significant correlations with the occurrence of proteinuria.

**Table 2:** Clinical characteristics and pharmacokinetic parameters of the patients in the toxicity study of proteinuria.

Characteristic	Proteinuria (*n* = 8)	NO proteinuria(*n* = 8)	P value*
Age (years)	53.1 ± 10.9	46.0 ± 14.8	0.291
BMI (kg/m^2^)	23.9 ± 3.4	23.4 ± 2.8	0.739
Gender			1.000
Male	4	4	
Female	4	4	
AUC_(0–t)_ (μg/L*h)	797.9 ± 257.1	782.8 ± 199.1	0.897
AUC_(0–∞)_ (μg/L*h)	1076.6 ± 318.3	1018.2 ± 264.6	0.696
C_max_ (μg/L)	7.84 ± 2.81	8.52 ± 2.79	0.632
Tumor types			0.647
Endometrial stromal tumor	*n* = 0	*n* = 1	
Adenoid cystic tumor	*n* = 2	*n* = 2	
Schwannoma	*n* =v2	*n* = 2	
Thyroid follicular tumor	*n* = 1	*n* = 0	
Hepatic carcinoma	*n* = 0	*n* = 1	
Corticosuprarenaloma	*n* = 0	*n* = 1	
Thymic carcinoma	*n* = 1	*n* = 0	
Medullary thyroid carcinoma	*n* = 1	*n* = 1	
Hemangiosarcoma	*n* = 1	*n* = 0	

*P values were calculated using bilateral t-test for age, BMI, AUC_(0–t)_, AUC_(0–∞)_ and C_max_ and using Chi^2^ test for gender and tumor types.

**Figure 6:**
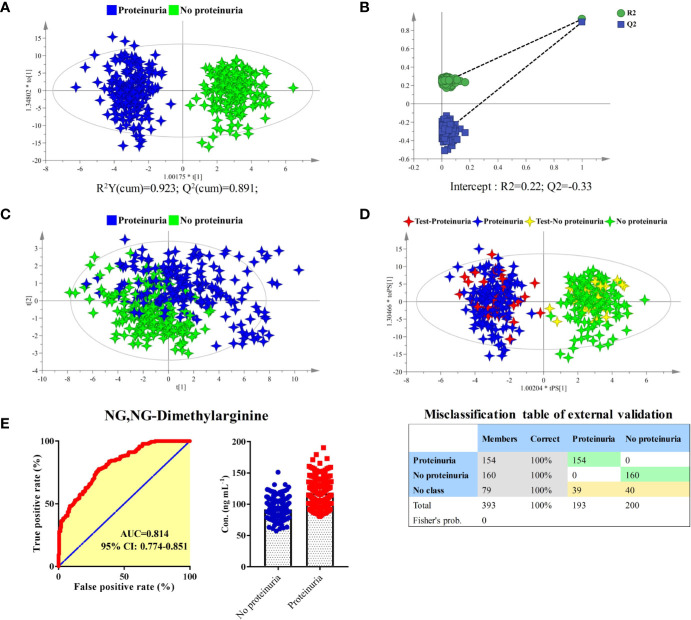
Identification of predictive biomarker associated with the AE of proteinuria after anlotinib administration. **(A)** OPLS-DA score plot of proteinuria and no proteinuria groups based on concentrations of all metabolites. **(B)** Random permutation test with 100 iterations. **(C)** PCA score plot of proteinuria and no proteinuria groups based on concentrations of the 15 metabolites with VIP values larger than 1.5. **(D)** External validation of the OPLS-DA model. Samples in the discovery set were designed as proteinuria or no proteinuria groups. While samples in the validation set were designed as test-proteinuria or test-no proteinuria groups. Both depended on whether proteinuria occurs. **(E)** ROC and box plot of NG, NG-dimethylarginine.

The longitudinal metabolomics data was employed for the discovery of potential biomarkers for the prediction of proteinuria after anlotinib administration. Due to the limited subject number and the relatively stable longitudinal metabolic fingerprint of the same subject in different time points of anlotinib treatment, metabolomics data from all the 25 time points was included in the statistical analysis to increase the reliability of the statistical results. The OPLS-DA was employed to discover the most obviously different metabolites between the proteinuria and no proteinuria groups, with result shown in **Figure 6A**. The subjects with AE of proteinuria were completely separated from the subjects without proteinuria. As shown in **Figure 6B**, no overfitting of the OPLS-DA model was observed through a random permutation test with 100 iterations. Significant different metabolic phenotypes existed between the proteinuria and no proteinuria groups. A total of 15 metabolites with VIP > 1.5 in OPLS-DA were screened out. Unsupervised PCA was conducted based on concentrations of these 15 metabolites, with score plot shown in **Figure 6C**. A moderate separation of the proteinuria and no proteinuria groups can be achieved in this unsupervised statistical method.

In order to carry out external validation of the OPLS-DA model, 80% of the total sample size was designed as the discovery set, and the other 20% was used for model validation. Based on the presence or absence of proteinuria, samples in the discovery set were classified as proteinuria or no proteinuria groups. As shown in **Figure 6D**, excellent OPLS-DA model was established based on samples from the discovery set. The established OPLS-DA model was further used for the discrimination of samples in the validation set. As shown in the figure, all the samples in the validation set can be distinguished with 100% accuracy based on the established OPLS-DA model.

To screen potential biomarkers associated with the AE of proteinuria, the 15 metabolites with VIP > 1.5 from OPLS-DA were further applied for bilateral t-test and ROC analysis. NG, NG-dimethylarginine was found to be the most obviously different metabolite between proteinuria and no proteinuria groups. The ROC curve and box plot of NG, NG-dimethylarginine were shown in **Figure 6E**. The AUC of NG, NG-dimethylarginine was 0.814 (95% CI: 0.774–0.851). It was the most promising biomarker for the prediction of proteinuria occurrence after anlotinib administration. In order to explore the earliest occurrence time point of the NG, NG-dimethylarginine difference, subjects with or without the AE of proteinuria were evaluated for their NG, NG-dimethylarginine levels in different time points. As shown in **Figure S3**, significant different (P < 0.05) concentrations of NG, NG-dimethylarginine can be observed between proteinuria and no proteinuria groups based on the time points of SH1, SH2 and SH4, indicating that NG, NG-dimethylarginine levels were different between the proteinuria and no proteinuria groups as early as 4 h after anlotinib administration. With more time points included, the difference of NG, NG-dimethylarginine levels between the proteinuria and no proteinuria groups became more and more obvious, and the AUC under the ROC curve was also increasing (**Figure S3**).

## Discussion

Many patients experienced little efficacy or even toxicity with prescribed drugs (14). It was estimated that over two million patients had serious adverse drug reactions requiring hospitalization, producing permanent disability, or leading to death in US hospitals in 1994 (25). In particular, for anti-tumor drugs, there are often a variety of AEs and significant individual differences in efficacy. Personalized medicine is the selection of medicines for subgroups or even individuals to maximize drug efficacy and minimize toxicity (14, 15). In the past decade, pharmacometabonomics has been successfully used in the development of new drugs, prediction of drug metabolism, efficacy, and toxicity (14, 15, 21). It has already become an important tool for precision medicine research. Pharmacometabonomics is practical to screen and follow longitudinally patients for certain types of therapy such as anti-tumor therapy. Thus the concept of longitudinal pharmacometabonomics was introduced. Longitudinal pharmacometabonomics is dealing with metabolic trajectories as opposed to static metabolic profiles. Much greater information with high quality can be achieved for each patient using longitudinal pharmacometabonomics.

In this study, the longitudinal pharmacometabonomics was used for the first time to predict malignant tumor patient responses to anlotinib therapy. Results showed that 38 metabolites (**Table S6**) were significantly upregulated attributing to anlotinib treatment. The most obviously disturbed metabolic pathways were aminoacyl-tRNA biosynthesis, alanine, aspartate and glutamate metabolism, steroid hormone biosynthesis, citrate cycle, tyrosine metabolism, and arginine and proline metabolism (**Table S4**). According to the report of Kelly and coworkers, dasatinib treatment caused significant metabolomics variations in the desmoid tumor cell line T219 (26). All the disturbed metabolites, including asparagine, aspartate, dimethylamine, glucose-1-phosphate, glutamate, glutathione, isoleucine, leucine, proline, uridine, and valine were significantly upregulated after dasatinib treatment. Another work reported by Teresa and coworkers demonstrated a significantly higher concentrations of glutamic acid, aspartic acid, serine, hydroxyproline, methionine, asparagine, malate, fumarate, and succinate in erlotinib-sensitive tumor tissue than erlotinib-insensitive tumor tissue (27). As with dasatinib and erlotinib, anlotinib also belongs to the class of tinib anti-cancer drugs. Anlotinib treatment significantly improved the plasma levels of amino acids and metabolites associated with energy metabolism, which was highly consistent with dasatinib and erlotinib treatment. Our study suggested that the upregulation of amino acid and metabolites related to energy metabolism was the metabolic phenotypes of anlotinib and other tinib anti-cancer drugs.

There is an urgent need to find appropriate biomarkers to accurately predict and monitor the early efficacy, toxicity, and drug resistance of Anlotinib. In the ALTER0303 trial, activated circulating endothelial cells (aCECs) were measured in patients receiving anlotinib or placebo (28, 29). However, there was no significant relationship between progression free survival (PFS) and aCEC min/baseline in patients receiving placebo. Further study demonstrated that there was no correlation between sensitizing endothelial growth factor receptor (EGFR) mutations and PFS in 27 patients (5.53 months *vs* 5.53 months, HR = 1.16, 95% CI 0.73–1.85, P = 0.495) (30). Similarly, the EGFR T790M mutation did not reflect the treatment efficacy of anlotinib in 17 patients with advanced NSCLC (5.53 months *vs* 5.53 months, HR = 1.35, 95% CI 0.75–2.41, P = 0.253) (30). Unfortunately, all these results achieved by pharmacogenomics study can hardly provide any accurate and sensitive biomarkers for efficacy and toxicity prediction of anlotinib.

Here, the OPLS-DA models were established for the predication of efficacy and toxicity of anlotinib based on the longitudinal pharmacometabonomics study. Both internal and external validation results indicated the excellent accuracy of the OPLS-DA models. The anti-tumor efficacy and occurrence of proteinuria after anlotinib administration can be predicted with 100% accuracy using the established OPLS-DA models. Further statistical analysis showed the glycodeoxycholic acid and glycocholic acid possessed the most excellent sensitivity and specificity in predicting the efficacy of anlotinib, with AUCs of ROC 0.847 (95% CI: 0.803–0.886) and 0.828 (95% CI: 0.781–0.868), respectively. Lower plasma concentrations of glycodeoxycholic acid and glycocholic acid indicated better efficacy of anlotinib. In addition, NG, NG-dimethylarginine was found to be the most promising biomarker for the prediction of proteinuria occurrence after anlotinib administration, with AUCs of ROC 0.814 (95% CI: 0.774–0.851). NG, NG-dimethylarginine is a metabolic by-product of continual protein modification processes in the cytoplasm of all human cells. It is created in protein methylation, a common mechanism of post-translational protein modification. NG, NG-dimethylarginine has been identified as a uremic toxin according to the European Uremic Toxin Wording Group (31).

This is the first study to use longitudinal pharmacometabonomics to explore biomarkers for prediction of efficacy and toxicity after anlotinib administration. Metabolomics data of plasma samples from all the 25 time points (18 time points of subject 008) were included in the biomarkers investigation procedures due to the relative stable metabolic fingerprint of the same subject at different time points. Metabolomics data of 393 samples from 16 subjects was finally applied to statistical analysis for biomarker investigation. The bias caused by small sample size can be significantly improved through this strategy. However, it is still necessary to use larger samples to verify whether the models and biomarkers established in this study can be used in the final clinical practice. Another limitation of this study was the absence of pre-dose metabolic phenotype. For the exploring of metabolic phenotype variation related to anlotinib treatment, the metabolomics data of 1 h (SH1) after the first anlotinib dosing was used instead of the baseline metabolomics data. Apart from the limitations of relative small sample size and the absence of baseline phenotype, this study pioneered the use of longitudinal pharmacometabonomics for predicting malignant tumor patient responses to anlotinib therapy.

## Conclusion

In summary, our study disclosed the metabolic phenotype variation after anlotinib treatment. In addition, the mathematical models and potential biomarkers were established for the predication of anlotinib efficacy and toxicity based on the longitudinal pharmacometabonomics study. The anti-tumor efficacy and occurrence of proteinuria after anlotinib treatment can be predicted with 100% accuracy using the established OPLS-DA models. Through further validation with a larger sample size, we believed that the established OPLS-DA models and potential biomarkers can be eventually applied to predict the efficacy and toxicity of anlotinib in clinic. The results achieved in this study demonstrated the broad prospects and values of longitudinal pharmacometabonomics in promoting the precision use of anti-tumor drugs.

## Data Availability Statement

The datasets presented in this study can be found in online repositories. The names of the repository/repositories and accession number(s) can be found below: FigShare, https://doi.org/10.6084/m9.figshare.13050797.

## Ethics Statement

The studies involving human participants were reviewed and approved by Ethics committee of Beijing Chao-Yang Hospital. Written informed consent for participation was not required for this study in accordance with the national legislation and the institutional requirements.

## Author Contributions

LL, ZA, and TH conceived and designed this study. YC, YS, and XW implemented the clinical trial and collected and plasma samples. TH, PD, PL, and ZA performed the experiments and interpreted the data. TH and ZA wrote and revised the manuscript. All authors contributed to the article and approved the submitted version.

## Funding

This study was supported by the following grants: National Science and Technology Major Projects for ‘Major New Drugs Innovation and Development’ (grant number 2017ZX09101001); National Natural Science Foundation of China (grant number 81903569); Beijing Municipal Natural Science Foundation (grant number 7204266).

## Conflict of Interest

Author XW was employed by the company Chia Tai Tianqing Pharmaceutical Group Co.

The remaining authors declare that the research was conducted in the absence of any commercial or financial relationships that could be construed as a potential conflict of interest.
