# Supplementary material for: Longitudinal Pharmacometabonomics for Predicting Malignant Tumor Patient Responses to Anlotinib Therapy: Phenotype, Efficacy, and Toxicity
Source: Front Oncol. 2020 Nov 12;10:548300. doi: 10.3389/fonc.2020.548300 (PMC7689013; doi:10.3389/fonc.2020.548300)
Supplement: Supplementary file 1 [file DataSheet_1.docx]

Supplementary Material

**Longitudinal pharmacometabonomics for predicting malignant tumor patient responses to anlotinib therapy: phenotype, efficacy and toxicity**

**Ting Hu^1#^, Zhuoling An^1#^, Yongkun Sun^2^, Xunqiang Wang^3^, Ping Du^1^, Pengfei Li^1^, Yihebali Chi^2*^, Lihong Liu^1*^**

^1^Beijing Chao-Yang Hospital, Capital Medical University, Beijing, China

^2^National Cancer Center, National Clinical Research Center for Cancer, Cancer Hospital, Chinese Academy of Medical Sciences and Peking Union Medical College, Beijing, China

^3^Chia Tai Tianqing Pharmaceutical Group Co., Nanjing, China

**Table S1.** Metabolites information, MRM parameters and quantification performance.

| **Metabolites** | **CAS NO.** | **Formula** | **MS polarity** | **Q1** | **Q3** | **DP** | **CE** | **LLOQ (ng mL^-1^)** | **Dynamic range** | ***r*** |
| --- | --- | --- | --- | --- | --- | --- | --- | --- | --- | --- |
| Choline | 62-49-7 | C5H14NO^+^ | Positive | 104.1 | 60 | 90 | 40 | 2 | 20-1000 | 0.9953 |
| Niacinamide | 98-92-0 | C6H6N2O | Positive | 123.1 | 80 | 70 | 30 | 1 | 20-2000 | 0.9949 |
| 1,3-Diaminopropane | 109-76-2 | C3H10N2 | Positive | 75 | 58 | 112 | 15 | 2 | 20-1000 | 0.9964 |
| Glycerol | 56-81-5 | C3H8O3 | Positive | 93 | 66 | 207 | 21 | 100 | 500-5000 | 0.9971 |
| L-Glutamine | 56-85-9 | C5H10N2O3 | Positive | 147 | 84 | 140 | 35 | 10 | 20-1000 | 0.9994 |
| L-Histidine | 71-00-1 | C6H9N3O2 | Positive | 156.1 | 110 | 70 | 20 | 1 | 2-1000 | 0.9945 |
| L-Proline | 147-85-3 | C5H9NO2 | Positive | 116 | 70 | 100 | 40 | 0.5 | 20-2000 | 0.9918 |
| L-Phenylalanine | 63-91-2 | C9H11NO2 | Positive | 166 | 120 | 90 | 18 | 0.2 | 2-2000 | 0.9961 |
| L-Glutamic acid | 56-86-0 | C5H9NO4 | Positive | 148.1 | 84 | 60 | 38 | 10 | 20-2000 | 0.9972 |
| L-isoleucine | 73-32-5 | C6H13NO2 | Positive | 132 | 86 | 50 | 14 | 0.1 | 20-2000 | 0.9946 |
| L-Tyrosine | 60-18-4 | C9H11NO3 | Positive | 182 | 136 | 120 | 19 | 2 | 20-5000 | 0.9992 |
| L-Cystine | 56-89-3 | C6H12N2O4S2 | Positive | 241 | 152 | 80 | 19 | 100 | 200-5000 | 0.9946 |
| L-Alanine | 56-41-7 | C3H7NO2 | Positive | 90 | 44 | 104 | 20 | 10 | 50-5000 | 0.9943 |
| HEPES | 7365-45-9 | C8H18N2O4S | Positive | 239.2 | 131 | 200 | 30 | 2 | 20-2000 | 0.9960 |
| Thiamine pyrophosphate | 154-87-0 | C12H19ClN4O7P2S | Positive | 425.2 | 122 | 120 | 40 | 100 | 500-5000 | 0.9968 |
| 5'-Methylthioadenosine | 2457-80-9 | C11H15N5O3S | Positive | 298.1 | 136 | 90 | 27 | 0.05 | 0.2-500 | 0.9969 |
| S-Adenosyl-L-methionine | 86867-01-8 | C15H25Cl3N6O5S | Positive | 399.4 | 298 | 100 | 12 | 20 | 200-5000 | 0.9944 |
| AMP | 61-19-8 | C10H14N5O7P | Positive | 348.1 | 136 | 80 | 70 | 1 | 20-5000 | 0.9993 |
| GMP | 7665-99-8 | C10H12N5O7P | Positive | 346.1 | 152 | 90 | 30 | 0.1 | 2-2000 | 0.9968 |
| dAMP | 653-63-4 | C10H14N5O6P | Positive | 332.1 | 136 | 60 | 22 | 0.5 | 50-5000 | 0.9984 |
| L-Carnosine | 305-84-0 | C9H14N4O3 | Positive | 227.3 | 122 | 100 | 19 | 20 | 100-5000 | 0.9973 |
| Homocysteine | 454-29-5 | C4H9NO2S | Positive | 136.1 | 90 | 60 | 15 | 2 | 20-5000 | 0.9948 |
| Guanosine | 118-00-3 | C10H13N5O5 | Positive | 284.2 | 152 | 60 | 20 | 0.2 | 0.5-2000 | 0.9969 |
| CMP | 63-37-6 | C9H14N3O8P | Positive | 324.1 | 112 | 60 | 25 | 2 | 50-5000 | 0.9961 |
| L-Aspartate | 56-84-8 | C4H7NO4 | Positive | 134 | 74 | 60 | 20 | 50 | 100-5000 | 0.9985 |
| DL 2:0 | 3040-38-8 | C9H17NO4 | Positive | 204.2 | 85 | 100 | 69 | 10 | 50-5000 | 0.9978 |
| L-Asparagine | 70-47-3 | C4H8N2O3 | Positive | 133.1 | 87 | 50 | 12 | 10 | 50-2000 | 0.9932 |
| L-Valine | 72-18-4 | C5H11NO2 | Positive | 118 | 72 | 115 | 14 | 1 | 100-5000 | 0.9964 |
| Histamine | 51-45-6 | C5H9N3 | Positive | 112 | 95 | 60 | 10 | 1 | 2-500 | 0.9944 |
| L-Lysine | 56-87-1 | C6H14N2O2 | Positive | 147.1 | 84 | 65 | 35 | 2 | 50-5000 | 0.9941 |
| Glycine | 56-40-6 | C2H5NO2 | Positive | 76 | 30 | 30 | 20 | 20 | 200-5000 | 0.9940 |
| L-Leucine | 61-90-5 | C6H13NO2 | Positive | 132.1 | 86 | 100 | 20 | 0.1 | 20-5000 | 0.9989 |
| trans-4-Hydroxy-L-proline | 51-35-4 | C5H9NO3 | Positive | 132.1 | 86 | 100 | 19 | 1 | 20-5000 | 0.9985 |
| Thymidine | 50-89-5 | C10H14N2O5 | Positive | 243.1 | 127 | 80 | 20 | 1 | 5-5000 | 0.9975 |
| L-Serine | 56-45-1 | C3H7NO3 | Positive | 106 | 60 | 55 | 15 | 2 | 20-2000 | 0.9956 |
| Nicotinate | 59-67-6 | C6H5NO2 | Positive | 124.1 | 80 | 120 | 30 | 10 | 20-2000 | 0.9946 |
| Ornithine | 3184-13-2 | C5H12N2O2 | Positive | 133.1 | 70 | 100 | 22 | 1 | 5-2000 | 0.9972 |
| L-Tryptophan | 73-22-3 | C11H12N2O2 | Positive | 205.2 | 188 | 100 | 14 | 0.1 | 0.5-2000 | 0.9984 |
| sn-Glycero-3-phosphocholine | 28319-77-9 | C8H20NO6P | Positive | 258.2 | 104 | 100 | 22 | 1 | 20-5000 | 0.9964 |
| GMP | 85-32-5 | C10H14N5O8P | Positive | 364.1 | 152.1 | 60 | 28 | 20 | 100-5000 | 0.9962 |
| Trimethylamine-N-oxide | 1184-78-7 | C3H9NO | Positive | 76.1 | 42 | 160 | 34 | 10 | 50-2000 | 0.9925 |
| NG,NG-Dimethylarginine | 30315-93-6 | C8H18N4O2 | Positive | 203.2 | 158 | 120 | 20 | 2 | 20-2000 | 0.9963 |
| L-Kynurenine | 2922-83-0 | C10H12N2O3 | Positive | 209.2 | 94 | 40 | 20 | 0.5 | 2-5000 | 0.9989 |
| N-Acetyl-L-Glutamine | 2490-97-3 | C7H12N2O4 | Positive | 189.2 | 130 | 35 | 23 | 1 | 5-2000 | 0.9965 |
| Adenine | 73-24-5 | C5H5N5 | Positive | 135.8 | 118.8 | 60 | 20 | 10 | 50-2000 | 0.9960 |
| Allantoin | 97-59-6 | C4H6N4O3 | Positive | 159.1 | 116 | 60 | 11 | 2 | 20-2000 | 0.9962 |
| Aminoisobutyrate | 62-57-7 | C4H9NO2 | Positive | 104.1 | 58 | 100 | 17 | 1 | 5-2000 | 0.9954 |
| Glycylglycine | 556-50-3 | C4H8N2O3 | Positive | 133.1 | 76 | 70 | 13 | 0.5 | 5-2000 | 0.9973 |
| γ-Aminobutanoate | 56-12-2 | C4H9NO2 | Positive | 104.1 | 58 | 100 | 17 | 0.5 | 5-2000 | 0.9942 |
| Betaine | 107-43-7 | C5H11NO2 | Positive | 118.1 | 43 | 100 | 55 | 20 | 100-5000 | 0.9940 |
| Hypoxanthine | 68-94-0 | C5H4N4O | Positive | 137.1 | 110 | 160 | 28 | 1 | 20-2000 | 0.8843 |
| Creatine | 57-00-1 | C4H9N3O2 | Positive | 132.1 | 90 | 135 | 27 | 2 | 20-1000 | 0.9954 |
| Cysteine | 52-90-4 | C3H7NO2S | Positive | 122.2 | 59 | 45 | 30 | 2 | 20-2000 | 0.9923 |
| DL-Pyroglutamic acid | 149-87-1 | C5H7NO3 | Positive | 130.1 | 84 | 70 | 18 | 1 | 200-5000 | 0.9980 |
| Hippurate | 495-69-2 | C9H9NO3 | Positive | 180.1 | 105 | 60 | 20 | 0.5 | 0.5-2000 | 0.9978 |
| Cystamine | 51-85-4 | C4H12N2S2 | Positive | 153.2 | 108 | 90 | 25 | 10 | 100-2000 | 0.9915 |
| DOPA | 59-92-7 | C9H11NO4 | Positive | 198.2 | 152 | 100 | 20 | 1 | 20-5000 | 0.9984 |
| Citrulline | 372-75-8 | C6H13N3O3 | Positive | 176.1 | 70 | 70 | 31 | 2 | 5-2000 | 0.9978 |
| cAMP | 60-92-4 | C10H12N5O6P | Positive | 330.1 | 136 | 110 | 33 | 0.1 | 0.5-2000 | 0.9966 |
| Inositol | 87-89-8 | C6H12O6 | Positive | 181.1 | 139 | 145 | 15 | 10 | 100-5000 | 0.9969 |
| Uracil | 66-22-8 | C4H4N2O2 | Positive | 113 | 70 | 140 | 20 | 2 | 50-2000 | 0.9958 |
| Pantothenate | 137-08-6 | C18H32CaN2O10 | Positive | 220.1 | 90 | 80 | 20 | 0.5 | 2-5000 | 0.9967 |
| 7-Methylxanthine | 552-62-5 | C6H6N4O2 | Positive | 167.1 | 124 | 100 | 28 | 0.5 | 2-2000 | 0.9977 |
| 2'-Deoxyadenosine | 16373-93-6 | C10H15N5O4 | Positive | 252.3 | 94 | 80 | 61 | 2 | 5-500 | 0.9952 |
| Pyridoxal 5'-phosphate | 41468-25-1 | C8H12NO7P | Positive | 248.2 | 150 | 100 | 27 | 0.2 | 20-2000 | 0.9963 |
| 5-Methyluridine | 1463-10-1 | C10H14N2O6 | Positive | 259.1 | 127 | 60 | 16 | 1 | 5-5000 | 0.9966 |
| 2'-Deoxycytidine | 951-77-9 | C9H13N3O4 | Positive | 228.1 | 112 | 50 | 20 | 0.2 | 5-2000 | 0.9951 |
| Deoxyuridine | 951-78-0 | C9H12N2O5 | Positive | 229.2 | 113 | 60 | 20 | 2 | 50-5000 | 0.9933 |
| Inosine | 58-63-9 | C10H12N4O5 | Positive | 269.1 | 137 | 60 | 23 | 0.2 | 5-1000 | 0.9990 |
| Carnitine | 541-15-1 | C7H15NO3 | Positive | 162.1 | 103 | 60 | 35 | 2 | 5-500 | 0.9909 |
| 4-Aminobenzoate | 150-13-0 | C7H7NO2 | Positive | 138.1 | 94 | 80 | 18 | 2 | 5-5000 | 0.9984 |
| Hydroxyproline | 51-35-4 | C5H9NO3 | Positive | 132.1 | 86 | 60 | 17 | 1 | 5-2000 | 0.9964 |
| Pyridoxine | 65-23-6 | C8H11NO3 | Positive | 170 | 134.2 | 60 | 20 | 0.2 | 2-1000 | 0.9918 |
| Cytosine | 71-30-7 | C4H5N3O | Positive | 112 | 95 | 60 | 20 | 1 | 2-1000 | 0.9976 |
| Dopamine | 51-61-6 | C8H11NO2 | Positive | 154.1 | 119 | 105 | 29 | 1 | 20-5000 | 0.9963 |
| Creatinine | 60-27-5 | C4H7N3O | Positive | 114.1 | 86 | 140 | 22 | 10 | 50-2000 | 0.9920 |
| Cytidine | 65-46-3 | C9H13N3O5 | Positive | 244.1 | 112 | 70 | 24 | 0.2 | 20-5000 | 0.9986 |
| N-Acetyl-D-glucosamine | 7512-17-6 | C8H15NO6 | Positive | 222.1 | 138 | 50 | 21 | 10 | 50-2000 | 0.9962 |
| 5-Methylcytidine | 2140-61-6 | C10H15N3O5 | Positive | 258.1 | 126 | 65 | 24 | 0.2 | 2-5000 | 0.9976 |
| N,N-Dimethylglycine | 1118-68-9 | C4H9NO2 | Positive | 104.1 | 58 | 50 | 21 | 0.5 | 5-500 | 0.9924 |
| Cysteamine | 60-23-1 | C2H7NS | Positive | 78.1 | 61 | 88 | 17 | 2 | 50-2000 | 0.9955 |
| Melatonin | 73-31-4 | C13H16N2O2 | Positive | 233.3 | 174 | 130 | 21 | 0.05 | 0.2-500 | 0.9960 |
| Uridine | 58-96-8 | C9H12N2O6 | Positive | 245.1 | 113 | 60 | 20 | 1 | 20-5000 | 0.9953 |
| 1,7-Dimethylxanthine | 611-59-6 | C7H8N4O2 | Positive | 181.1 | 124 | 90 | 27 | 0.1 | 0.5-2000 | 0.9957 |
| Homocystine | 626-72-2 | C8H16N2O4S2 | Positive | 269.3 | 136 | 90 | 14 | 1 | 20-5000 | 0.9941 |
| (±)-Epinephrine | 51-43-4 | C9H13NO3 | Positive | 184.2 | 107 | 100 | 30 | 10 | 100-5000 | 0.9903 |
| N-Acetylornithine | 6205-08-9 | C7H14N2O3 | Positive | 175 | 69.8 | 60 | 30 | 1 | 5-2000 | 0.9968 |
| Cobalamin | 68-19-9 | C63H88CoN14O14P | Positive | 678.8 | 147 | 230 | 65 | 0.5 | 2-5000 | 0.9975 |
| 3-Methylxanthine | 1076-22-8 | C6H6N4O2 | Positive | 167.1 | 124 | 100 | 24 | 0.5 | 0.5-5000 | 0.9960 |
| L-Arginine | 74-79-3 | C6H14N4O2 | Positive | 175 | 69.8 | 60 | 30 | 2 | 5-2000 | 0.9968 |
| L-Threonine | 72-19-5 | C4H9NO3 | Positive | 120.1 | 74 | 31 | 15 | 2 | 5-2000 | 0.9936 |
| Oxidized glutathione | 27025-41-8 | C20H32N6O12S2 | Positive | 613.1 | 230.8 | 180 | 48 | 20 | 100-5000 | 0.9940 |
| L-homoserine | 16504-56-6 | C4H9NO3 | Positive | 120 | 60 | 100 | 23 | 20 | 50-2000 | 0.9944 |
| DL 8:0 | 25243-95-2 | C15H29NO4 | Positive | 288.2 | 85 | 60 | 20 | 0.05 | 0.2-500 | 0.9951 |
| L-Cystathionine | 56-88-2 | C7H14N2O4S | Positive | 223.2 | 134 | 100 | 21 | 0.5 | 20-5000 | 0.9918 |
| normetanephrine | 1011-74-1 | C9H14ClNO3 | Positive | 184.2 | 134 | 80 | 24 | 10 | 100-2000 | 0.9959 |
| Ala-Gly | 3695-73-6 | C5H10N2O3 | Positive | 147.1 | 90 | 100 | 19 | 10 | 20-2000 | 0.9968 |
| Adenosine | 131-99-7 | C10H13N5O4 | Positive | 268.1 | 136 | 70 | 30 | 0.5 | 0.2-500 | 0.9928 |
| DCTP | 2056-98-6 | C9H16N3O13P3 | Positive | 468.1 | 395 | 60 | 15 | 100 | 500-5000 | 0.9998 |
| Putrescine | 110-60-1 | C4H12N2 | Positive | 89.1 | 72 | 100 | 23 | 100 | 200-5000 | 0.9933 |
| Spermidine | 124-20-9 | C7H19N3 | Positive | 146.2 | 112 | 100 | 13 | 10 | 50-2000 | 0.9906 |
| Spermine | 71-44-3 | C10H26N4 | Positive | 203.2 | 112.1 | 60 | 29 | 10 | 50-5000 | 0.9976 |
| Thiamine | 70-16-6 | C12H17N4OS^+^ | Positive | 265.3 | 122 | 100 | 52 | 2 | 2-200 | 0.9946 |
| Aldosterone | 52-39-1 | C21 H28 O5 | Positive | 361.2 | 343 | 170 | 26 | 2 | 2-5000 | 0.9937 |
| 11-Deoxycortisol | 152-58-9 | C21 H30 O4 | Positive | 347.2 | 97 | 170 | 33 | 0.2 | 0.5-2000 | 0.9964 |
| 11-Deoxycorticosterone | 64-85-7 | C21 H30 O3 | Positive | 331.2 | 97 | 170 | 27 | 0.5 | 0.5-2000 | 0.9961 |
| Corticosterone | 50-22-6 | C21 H30 O4 | Positive | 347.2 | 121.1 | 100 | 33 | 0.5 | 2-2000 | 0.9954 |
| Dehydroepiandrosterone | 53-43-0 | C19 H28 O2 | Positive | 289.2 | 85 | 100 | 27 | 0.2 | 0.2-500 | 0.9953 |
| Cortisol | 50-23-7 | C21 H30 O5 | Positive | 363.2 | 121 | 180 | 32 | 0.2 | 0.5-2000 | 0.9975 |
| Dihydrotestosterone | 521-18-6 | C19H30O2 | Positive | 291.2 | 145 | 70 | 23 | 10 | 20-5000 | 0.9975 |
| Androstenedione | 63-05-8 | C19H26O2 | Positive | 287.2 | 97 | 170 | 30 | 0.2 | 0.2-1000 | 0.9923 |
| Testosterone | 58-22-0 | C19H28O2 | Positive | 289.2 | 109 | 100 | 32 | 0.2 | 0.5-1000 | 0.9964 |
| 4-Pyridoxic acid | 82-82-6 | C8H9NO4 | Positive | 184 | 148 | 60 | 40 | 0.1 | 2-1000 | 0.9959 |
| Reduced glutathione | 70-18-8 | C10H17N3O6S | Positive | 308 | 179 | 150 | 18 | 2 | 50-5000 | 0.9976 |
| Lactose | 63-42-3 | C12H22O11 | Negative | 341 | 89 | -120 | -25 | 10 | 50-2000 | 0.9951 |
| D-Gluconic acid | 133-42-6 | C6H12O7 | Negative | 195 | 129 | -80 | -27 | 2 | 20-5000 | 0.9949 |
| Taurine | 107-35-7 | C2H7NO3S | Negative | 124 | 80 | -100 | -32 | 0.1 | 2-2000 | 0.9967 |
| Fructose | 57-48-7 | C6H12O6 | Negative | 179.1 | 89 | -90 | -13 | 2 | 20-1000 | 0.9960 |
| Ascorbic acid | 50-81-7 | C6H8O6 | Negative | 174.9 | 86.8 | -60 | -20 | 10 | 500-5000 | 0.9974 |
| Oxoacetic Acid | 298-12-4 | C2H2O3 | Negative | 73 | 45 | -120 | -11 | 20 | 200-5000 | 0.9971 |
| Glucose | 50-99-7 | C6H12O6 | Negative | 179.1 | 89 | -90 | -13 | 2 | 20-1000 | 0.9944 |
| MOPS | 1132-61-2 | C7H15NO4S | Negative | 207.8 | 79.9 | -60 | -30 | 0.05 | 0.5-2000 | 0.9982 |
| Xylitol | 87-99-0 | C5H12O5 | Negative | 151 | 89 | -100 | -16 | 2 | 20-1000 | 0.9974 |
| Succinate | 150-90-3 | C4H4Na2O4 | Negative | 117 | 73 | -70 | -15 | 2 | 20-5000 | 0.9977 |
| DCMP | 13085-50-2 | C9H12N3Na2O7P | Negative | 306 | 79 | -120 | -50 | 0.2 | 20-5000 | 0.9970 |
| Pyridoxal phosphate | 853645-22-4 | C8H10NO6P | Negative | 246 | 79 | -120 | -60 | 2 | 5-5000 | 0.9961 |
| Thymidine 5′-monophosphate (DTMP) | 33430-62-5 | C10H13N2Na2O8P | Negative | 321.1 | 79 | -70 | -60 | 2 | 2-5000 | 0.9969 |
| Phosphoethanolamine | 1071-23-4 | C2H8NO4P | Negative | 140 | 79 | -80 | -30 | 0.5 | 5-1000 | 0.9996 |
| D-Glucosamine 6-phosphate | 3616-42-0 | C6H14NO8P | Negative | 258.1 | 79 | -120 | -120 | 1 | 5-1000 | 0.9982 |
| DL-Glyceraldehyde 3-phosphate | 591-59-3 | C3H7O6P | Negative | 169 | 79 | -110 | -45 | 10 | 100-5000 | 0.9957 |
| Urate | 69-93-2 | C5H4N4O3 | Negative | 167 | 124 | -190 | -20 | 0.5 | 50-2000 | 0.9937 |
| Pyruvate | 127-17-3 | C3H4O3 | Negative | 87 | 32 | -40 | -13 | 20 | 200-5000 | 0.9948 |
| α-Keto-glutarate | 328-50-7 | C5H6O5 | Negative | 145 | 101 | -100 | -18 | 100 | 500-5000 | 0.9900 |
| Thymine | 65-71-4 | C5H6N2O2 | Negative | 125 | 42 | -100 | -32 | 1 | 2-5000 | 0.9984 |
| Fumarate | 110-17-8 | C4H4O4 | Negative | 115 | 71 | -40 | -11 | 10 | 100-5000 | 0.9980 |
| L-Gulono-1,4-lactone | 1128-23-0 | C6H10O6 | Negative | 177 | 89 | -120 | -17 | 10 | 20-2000 | 0.9968 |
| Xanthine | 69-89-6 | C5H4N4O2 | Negative | 151.1 | 108 | -140 | -22 | 10 | 20-5000 | 0.9957 |
| Biotin | 58-85-5 | C10H16N2O3S | Negative | 243.2 | 200 | -120 | -21 | 0.2 | 0.5-5000 | 0.9978 |
| Benzoate | 65-85-0 | C7H6O2 | Negative | 120.9 | 77.1 | -60 | -10 | 100 | 100-2000 | 0.9936 |
| Mevalonate | 150-97-0 | C6H12O4 | Negative | 147.1 | 59 | -110 | -37 | 100 | 500-5000 | 0.9965 |
| Orotate | 65-86-1 | C5H4N2O4 | Negative | 155 | 111 | -50 | -17 | 10 | 100-5000 | 0.9975 |
| N-acetyl-L-glutamate | 1188-37-0 | C7H11NO5 | Negative | 188 | 102 | -40 | -24 | 1 | 5-5000 | 0.9976 |
| 2-Oxoglutarate | 328-50-7 | C5H6O5 | Negative | 145.1 | 101 | -90 | -17 | 100 | 200-5000 | 0.9976 |
| L-(-)-Malic acid | 97-67-6 | C4H6O5 | Negative | 133 | 115 | -70 | -16 | 10 | 20-2000 | 0.9923 |
| Phenylacetylglycine | 500-98-1 | C10H11NO3 | Negative | 192 | 74 | -60 | -10 | 0.1 | 0.5-5000 | 0.9978 |
| L-Cysteic acid | 23537-25-9 | C3H9NO6S | Negative | 168.1 | 81 | -100 | -56 | 2 | 20-2000 | 0.9968 |
| 4-hydroxyphenylpyruvate | 156-39-8 | C9H8O4 | Negative | 179.2 | 107 | -45 | -12 | 10 | 50-5000 | 0.9941 |
| D-glucose 6-phosphate | 56-73-5 | C6H13O9P | Negative | 258.8 | 96.8 | -60 | -10 | 10 | 100-2000 | 0.9992 |
| 3',5'-Cyclic AMP | 60-92-4 | C10H12N5O6P | Negative | 328 | 134 | -60 | -30 | 0.02 | 0.2-2000 | 0.9964 |
| N-Acetylneuraminate | 131-48-6 | C11H19NO9 | Negative | 308.2 | 170 | -70 | -19 | 2 | 20-5000 | 0.9967 |
| NADH | 58-68-4 | C21H29N7O14P2 | Negative | 663.2 | 79 | -120 | -128 | 10 | 100-5000 | 0.9968 |
| Folate | 59-30-3 | C19H19N7O6 | Negative | 440.1 | 311 | -120 | -33 | 1 | 2-2000 | 0.9979 |
| Sucrose | 57-50-1 | C12H22O11 | Negative | 341 | 89 | -120 | -25 | 10 | 100-2000 | 0.9967 |
| D-Fructose 6-phosphate | 643-13-0 | C6H13O9P | Negative | 259 | 79 | -80 | -60 | 2 | 20-5000 | 0.9970 |
| L-Lactic Acid | 79-33-4 | C3H6O3 | Negative | 89 | 43 | -100 | -25 | 20 | 200-5000 | 0.9990 |
| Xanthosine | 146-80-5 | C10H12N4O6 | Negative | 283.1 | 108 | -100 | -50 | 0.1 | 0.5-5000 | 0.9993 |
| Lipoate | 1200-22-2 | C8H14O2S2 | Negative | 205 | 170.8 | -60 | -10 | 0.1 | 0.5-5000 | 0.9951 |
| UDP-glucose | 133-89-1 | C15H24N2O17P2 | Negative | 565 | 79 | -150 | -120 | 50 | 50-5000 | 0.9946 |
| D-Glucose 1-phosphate | 59-56-3 | C6H13O9P | Negative | 259 | 79 | -120 | -120 | 2 | 20-5000 | 0.9989 |
| D-Ribose | 50-69-1 | C5H10O5 | Negative | 149 | 89 | -110 | -10 | 10 | 100-2000 | 0.9917 |
| Maleate | 110-16-7 | C4H4O4 | Negative | 115 | 71 | -100 | -26 | 20 | 20-5000 | 0.9980 |
| N-Acetylaspartate | 3106-85-2 | C11H16N2O8 | Negative | 303.2 | 96 | -80 | -54 | 2 | 20-5000 | 0.9959 |
| 1-Methyluric acid | 708-79-2 | C6H6N4O3 | Negative | 181 | 138 | -80 | -20 | 0.2 | 20-5000 | 0.9941 |
| IMP | 352195-40-5 | C10H13N4O8P | Negative | 347 | 79 | -120 | -80 | 2 | 20-5000 | 0.9953 |
| D-Erythrose 4-phosphate | 585-18-2 | C4H9O7P | Negative | 199 | 97 | -55 | -15 | 10 | 100-5000 | 0.9981 |
| Dihydrofolate | 4033-27-6 | C19H21N7O6 | Negative | 442.1 | 176 | -110 | -35 | 0.5 | 5-5000 | 0.9982 |
| Estriol E3 | 50-27-1 | C18 H24 O3 | Negative | 287 | 143 | -190 | -70 | 100 | 500-5000 | 0.9983 |
| 17-hydroxypregnenolone | 387-79-1 | C21 H32 O3 | Negative | 331 | 287 | -150 | -26 | 100 | 100-5000 | 0.9986 |
| Estrone E1 | 53-16-7 | C18 H22 O2 | Negative | 269 | 145 | -180 | -48 | 100 | 500-5000 | 0.9957 |
| Pregnenolone | 145-13-1 | C21 H32 O2 | Negative | 315.2 | 282.2 | -230 | -30 | 2 | 5-5000 | 0.9959 |
| UMP | 3387-36-8 | C9H13N2O9P | Negative | 323 | 79 | -100 | -80 | 100 | 100-5000 | 0.9973 |
| FA 12:0 | 143-07-7 | C12H24O2 | Negative | 199.2 | 199.2 | -100 | -10 | 2 | 200-5000 | 0.9948 |
| FA 14:0 | 544-63-8 | C14H28O2 | Negative | 227.2 | 227.2 | -100 | -10 | 2 | 200-5000 | 0.9940 |
| FA 16:4 | 29259-52-7 | C16H24O2 | Negative | 247.2 | 247.2 | -100 | -10 | 1 | 200-5000 | 0.9983 |
| FA 16:1 | 373-49-9 | C16H30O2 | Negative | 253.2 | 253.2 | -100 | -10 | 1 | 200-5000 | 0.9981 |
| FA 16:0 | 57-10-3 | C16H32O2 | Negative | 255.2 | 255.2 | -100 | -30 | 1 | 10-2000 | 0.9930 |
| FA 17:1 | 29743-97-3 | C17H32O2 | Negative | 267.2 | 267.2 | -100 | -10 | 1 | 10-2000 | 0.9912 |
| FA 17:0 | 506-12-7 | C17H34O2 | Negative | 269.2 | 269.2 | -100 | -10 | 1 | 10-2000 | 0.9998 |
| FA 18:4 | 20290-75-9 | C18H28O2 | Negative | 275.2 | 275.2 | -100 | -10 | 1 | 10-2000 | 0.9907 |
| FA 18:3-α | 463-40-1 | C18H30O2 | Negative | 277.2 | 277.2 | -100 | -30 | 1 | 10-2000 | 0.9956 |
| FA 18:3-γ | 506-26-3 | C18H30O2 | Negative | 277.2 | 277.2 | -100 | -10 | 1 | 10-2000 | 0.9945 |
| FA 18:2 | 60-33-3 | C18H32O2 | Negative | 279.2 | 279.2 | -100 | -30 | 1 | 10-2000 | 0.9989 |
| FA 18:1 | 112-80-1 | C18H34O2 | Negative | 281.2 | 281.2 | -100 | -30 | 1 | 10-2000 | 0.9912 |
| FA 18:0 | 57-11-4 | C18H36O2 | Negative | 283.2 | 283.2 | -100 | -30 | 1 | 10-2000 | 0.9934 |
| FA 20:5 | 10417-94-4 | C20H30O2 | Negative | 301.2 | 301.2 | -100 | -10 | 1 | 10-2000 | 0.9923 |
| FA 20:4 | 506-32-1 | C20H32O2 | Negative | 303.2 | 303.2 | -100 | -30 | 1 | 10-2000 | 0.9967 |
| FA 20:3 | 1783-84-2 | C20H34O2 | Negative | 305.2 | 305.2 | -100 | -10 | 1 | 10-2000 | 0.9978 |
| FA 20:2 | 135498-07-6 | C20H36O2 | Negative | 307.2 | 307.2 | -100 | -10 | 1 | 10-2000 | 0.9932 |
| FA 20:1 | 62322-84-3 | C20H38O2 | Negative | 309.2 | 309.2 | -100 | -10 | 1 | 10-2000 | 0.9954 |
| FA 20:0 | 506-30-9 | C20H40O2 | Negative | 311.2 | 311.2 | -100 | -10 | 1 | 10-2000 | 0.9962 |
| FA 22:6 | 6217-54-5 | C22H32O2 | Negative | 327.2 | 327.2 | -100 | -30 | 1 | 10-2000 | 0.9981 |
| FA 22:5- n3 | 24880-45-3 | C22H34O2 | Negative | 329.2 | 329.2 | -100 | -10 | 1 | 10-2000 | 0.9916 |
| FA 22:5- n6 | 25182-74-5 | C22H34O2 | Negative | 329.2 | 329.2 | -100 | -10 | 1 | 10-2000 | 0.9930 |
| FA 22:4 | - | C22H36O2 | Negative | 331.2 | 331.2 | -100 | -10 | 1 | 10-2000 | 0.9985 |
| FA 22:3-iso1 | 28845-86-5 | C22H38O2 | Negative | 333.2 | 333.2 | -100 | -10 | 1 | 10-2000 | 0.9980 |
| FA 22:2 | 17735-98-7 | C22H40O2 | Negative | 335.2 | 335.2 | -100 | -10 | 1 | 10-2000 | 0.9970 |
| FA 22:1 | 112-86-7 | C22H42O2 | Negative | 337.2 | 337.2 | -100 | -10 | 1 | 10-2000 | 0.9932 |
| FA 22:0 | 112-85-6 | C22H44O2 | Negative | 339.2 | 339.2 | -100 | -10 | 1 | 10-2000 | 0.9940 |
| Dehydrolithocholic acid | 1553-56-6 | C24H38O3 | Negative | 373.3 | 373.3 | -150 | -20 | 0.05 | 20-500 | 0.9995 |
| Lithocholic acid | 434-13-9 | C24H40O3 | Negative | 375.3 | 375.3 | -150 | -15 | 0.05 | 2-2000 | 0.9986 |
| Alloisolithocholic acid | 2276-93-9 | C24H40O3 | Negative | 375.3 | 375.3 | -150 | -20 | 0.1 | 5-5000 | 0.9965 |
| Isolithocholic acid | 1534-35-6 | C24H40O3 | Negative | 375.3 | 375.3 | -150 | -20 | 0.1 | 5-5000 | 0.9959 |
| Nordeoxycholic acid | 53608-86-9 | C23H38O4 | Negative | 377.3 | 377.3 | -150 | -20 | 0.1 | 0.5-2000 | 0.9978 |
| 5α-cholanic acid-3,6-dione | 6929-22-2 | C24 H36 O4 | Negative | 387.3 | 387.3 | -150 | -15 | 0.1 | 5-2000 | 0.9958 |
| 3,7-Diketocholanic acid | 859-97-2 | C24 H36 O4 | Negative | 387.3 | 387.3 | -150 | -20 | 0.2 | 2-2000 | 0.9973 |
| Apocholic acid | 641-81-6 | C24H38O4 | Negative | 389.3 | 389.3 | -150 | -20 | 0.1 | 20-5000 | 0.9950 |
| 5α-cholanic acid-3α-ol-6-one | 10573-17-8 | C24 H38 O4 | Negative | 389.3 | 389.3 | -150 | -20 | 0.1 | 0.5-2000 | 0.9946 |
| 7-Ketolithocholic acid | 4651-67-6 | C24 H38 O4 | Negative | 389.3 | 389.3 | -150 | -20 | 0.1 | 2-2000 | 0.9981 |
| 12-Ketolithocholic acid | 5130-29-0 | C24 H38 O4 | Negative | 389.3 | 389.3 | -150 | -20 | 0.1 | 0.2-2000 | 0.9973 |
| Isodeoxycholic acid | 566-17-6 | C24H40O4 | Negative | 391.3 | 391.3 | -150 | -15 | 0.05 | 0.5-2000 | 0.9950 |
| Deoxycholic acid | 83-44-3 | C24H40O4 | Negative | 391.3 | 391.3 | -150 | -20 | 0.05 | 2-1000 | 0.9959 |
| Ursodeoxycholic acid | 128-13-2 | C24H40O4 | Negative | 391.3 | 391.3 | -150 | -20 | 0.05 | 0.5-2000 | 0.9950 |
| Hyodeoxycholic acid | 83-49-8 | C24H40O4 | Negative | 391.3 | 391.3 | -150 | -20 | 0.05 | 0.5-5000 | 0.9971 |
| Dehydrocholic acid | 81-23-2 | C24H34O5 | Negative | 401.3 | 401.3 | -150 | -20 | 0.05 | 2-5000 | 0.9984 |
| Dioxolithocholic acid | 517-33-9 | C24H36O5 | Negative | 403.3 | 403.3 | -150 | -20 | 0.1 | 5-5000 | 0.9985 |
| 6,7-Diketolithocholic acid | - | C24H36O5 | Negative | 403.3 | 403.3 | -150 | -20 | 0.1 | 2-2000 | 0.9954 |
| 12-Ketochenodeoxycholic acid | 2458-08-4 | C24H38O5 | Negative | 405.3 | 405.3 | -260 | -20 | 0.05 | 2-2000 | 0.9965 |
| Cholic acid | 81-25-4 | C24H40O5 | Negative | 407.3 | 407.3 | -150 | -20 | 0.1 | 0.5-2000 | 0.9980 |
| Glycoursocholanic acid | - | C26H43NO3 | Negative | 416.3 | 416.3 | -150 | -20 | 0.1 | 20-500 | 0.9970 |
| Glycodeoxycholic acid | 360-65-6 | C26H43NO5 | Negative | 448.3 | 448.3 | -150 | -15 | 0.05 | 2-2000 | 0.9963 |
| Glycoursodeoxycholic acid | 64480-66-6 | C26H43NO5 | Negative | 448.3 | 448.3 | -150 | -15 | 0.05 | 0.5-2000 | 0.9976 |
| Glycochenodeoxycholic acid | 640-79-9 | C26H43NO5 | Negative | 448.3 | 448.3 | -150 | -20 | 0.1 | 0.2-5000 | 0.9959 |
| Glycohyodeoxycholic acid | 13042-33-6 | C26H43NO5 | Negative | 448.3 | 448.3 | -150 | -20 | 0.05 | 0.5-2000 | 0.9970 |
| Glycodehydrocholic acid | - | C26H37NO6 | Negative | 458.3 | 458.3 | -150 | -15 | 0.05 | 2-5000 | 0.9968 |
| Glycocholic acid | 475-31-0 | C26H43NO6 | Negative | 464.3 | 464.3 | -150 | -15 | 0.05 | 0.5-2000 | 0.9966 |
| Taurolithocholic acid | - | C26H45NO5S | Negative | 482.5 | 80 | -150 | -110 | 0.02 | 0.2-5000 | 0.9968 |
| Tauroursodeoxycholic acid/Taurohyodeoxycholic acid | 14605-22-2 | C26H45NO6S | Negative | 498.3 | 80 | -150 | -120 | 0.02 | 0.2-2000 | 0.9967 |
| Taurodeoxycholic acid | 516-50-7 | C26H45NO6S | Negative | 498.3 | 80 | -150 | -120 | 0.02 | 0.2-2000 | 0.9980 |
| Taurochenodeoxycholic acid | 516-35-8 | C26H45NO6S | Negative | 498.5 | 80 | -150 | -120 | 0.02 | 0.2-2000 | 0.9976 |
| Taurodehydrocholic acid | 517-37-3 | C26H39NO7S | Negative | 508.5 | 80 | -150 | -120 | 0.02 | 0.2-5000 | 0.9987 |
| Taurocholic acid | 81-24-3 | C26H45NO7S | Negative | 514.5 | 80 | -150 | -140 | 0.02 | 0.2-5000 | 0.9982 |

**Table S2.** Blood concentrations (ng mL^-1^) of anlotinib at different time points.

| **Anlotinib treatment** | **Time points** | **Time（d）** | **Subject 001** | **Subject 002** | **Subject 003** | **Subject 004** | **Subject 005** | **Subject 006** | **Subject 007** | **Subject 008** | **Subject 009** | **Subject 010** | **Subject 011** | **Subject 012** | **Subject 013** | **Subject 014** | **Subject 015** | **Subject 016** |
| --- | --- | --- | --- | --- | --- | --- | --- | --- | --- | --- | --- | --- | --- | --- | --- | --- | --- | --- |
| **Single-dose study** | Baseline | 0 | BLQ * | BLQ | BLQ | BLQ | BLQ | BLQ | BLQ | BLQ | BLQ | BLQ | BLQ | BLQ | BLQ | BLQ | BLQ | BLQ |
|  | SH1 | 0.0417 | 2.23 | 2.64 | 3.65 | 1.88 | 4.39 | 0.941 | 0.993 | 4.86 | 3.88 | 1.39 | 7.8 | 2.66 | 3.04 | 2.42 | 1.65 | 1.22 |
|  | SH2 | 0.0833 | 5.57 | 6.46 | 5.29 | 3.95 | 6.69 | 2.08 | 1.32 | 10.3 | 5.33 | 4.47 | 11 | 10.4 | 7.13 | 11.4 | 4.18 | 4.12 |
|  | SH4 | 0.17 | 4.62 | 6.72 | 10.7 | 4.31 | 6.96 | 4.1 | 2.1 | 7.92 | 4.23 | 4.78 | 9.24 | 10.4 | 7.38 | 6.07 | 5.53 | 9.4 |
|  | SH8 | 0.33 | 5.4 | 4.36 | 5.99 | 7.79 | 7.53 | 4.35 | 3.19 | 10.9 | 4.47 | 5.75 | 6.55 | 10.6 | 6.11 | 7.67 | 6.03 | 7.13 |
|  | SH11 | 0.46 | 3.99 | 4.47 | 5.77 | 6.3 | 10.6 | 4.41 | 2.91 | 8.88 | 4.94 | 6.86 | 5.77 | 9.17 | 4.77 | 7.68 | 5.39 | 8.61 |
|  | SH24 | 1 | 3.91 | 4.3 | 5.62 | 9.47 | 9.28 | 3.48 | 3.63 | 11.8 | 4.42 | 6.59 | 4.44 | 8.56 | 6.52 | 5.83 | 3.96 | 9.06 |
|  | SH48 | 2 | 3.34 | 3.54 | 4.51 | 5.23 | 8.01 | 3.14 | 3.09 | 7.45 | 4.11 | 4.48 | 3.38 | 6.35 | 4.88 | 4.61 | 3.24 | 5.68 |
|  | SH72 | 3 | 3.02 | 3.61 | 3.95 | 3.35 | 6.39 | 2.21 | 2.25 | 6.38 | 3.33 | 3.48 | 3.06 | 5.58 | 3.86 | 3.51 | 4.41 | 5.19 |
|  | SH120 | 5 | 2.07 | 3.04 | 2.97 | 3.06 | 3.48 | 1.83 | 1.98 | 4.97 | 2.81 | 2.63 | 2.61 | 2.82 | 3.12 | 2.24 | 1.7 | 3.65 |
|  | SH168 | 7 | 1.92 | 2.04 | 2.63 | 1.84 | 2.59 | 1.42 | 1.32 | 2.92 | 2.13 | 1.88 | 2.06 | 2.14 | 2.96 | 1.37 | 1.64 | 2.97 |
|  | SH240 | 10 | 1.28 | 1.3 | 1.8 | 1.32 | 1.65 | 1.13 | 1 | 2.21 | 1.37 | 1.4 | 1.35 | 0.97 | 2.03 | 0.947 | 1.1 | 2.06 |
| **First cycle of multiple-dose study** | C1D1 | 11 | 5.84 | 5.22 | 6.55 | 7.61 | 8.8 | 4.62 | 4.51 | 14.5 | 7.14 | 5.79 | 5.85 | 7.69 | 7.94 | 5.57 | 4.41 | 12.7 |
|  | C1D4 | 14 | 20.4 | 15.9 | 23.9 | 16.7 | 30.7 | 11.7 | 13.8 | 35.5 | 18.3 | 13.8 | 17.6 | 12.4 | 23.3 | 16.1 | 13.8 | 33.2 |
|  | C1D7 | 17 | 28 | 22.6 | 38.4 | 28.3 | 55.3 | 18.8 | 20.2 | 60.6 | 25.7 | 27.4 | 28.9 | 26 | 32.1 | 27.6 | 16.5 | 50.5 |
|  | C1D10 | 20 | 39 | 34.3 | 50 | 37.3 | 64.4 | 22.2 | 27.4 | 80.4 | 33 | 35 | 35.3 | 26.6 | 41.9 | 45.8 | 19.6 | 61.4 |
|  | C1D14 | 24 | 43.1 | 41 | 50.1 | 53.1 | 83.8 | 21.1 | 32.1 | 121 | 48.9 | 34.2 | 38 | 23.5 | 48.3 | 47.2 | 26 | 83.9 |
|  | C1D18 | 27 | 33.2 | 29.4 | 37.9 | 28.2 | 56.6 | 14.2 | 22 | 76.3 | 32.4 | 37.4 | 23.3 | 10.6 | 33.9 | 24 | 15.2 | 50.9 |
| **Second cycle of multiple-dose study** | C2D1-B | 31 | 17.7 | 17.5 | 24.1 | 13.8 | 25 | 8.06 | 15.2 | 44 | 17 | 21.3 | 15.4 | 5.05 | 20.2 | 13.5 | 11.1 | 28.5 |
|  | C2D1-A | 32 | 22.5 | 24.5 | 24.7 | 20.1 | 33.7 | 12.8 | 20.1 | NS | 21.3 | 29.4 | 18.1 | 9.72 | 29 | 17.4 | 15.3 | 41 |
|  | C2D4 | 35 | 30.8 | 30.5 | 35.9 | 32.2 | 56.4 | 19.6 | 32.6 | NS | 31.1 | 39.9 | 28 | 16.8 | 34.1 | 27.5 | 19 | 61.5 |
|  | C2D7 | 38 | 37.8 | 33.7 | 37.9 | 32.9 | 62.9 | 21.2 | 41.8 | NS | 38.8 | 45.8 | 30.8 | 18.4 | 40.4 | 34.1 | 26 | 76.7 |
|  | C2D10 | 41 | 38.2 | 43.4 | 47 | 41.5 | 71.6 | 22.9 | 57.4 | NS | 44.2 | 47.4 | 34.4 | 14.6 | 44.8 | 37.7 | 32.1 | 78.4 |
|  | C2D14 | 45 | 42.2 | 50 | 54.3 | 56.2 | 101 | 38.1 | 55.2 | NS | 40.4 | 44 | 32 | 22.1 | 45.1 | 42.2 | 36.1 | 73.9 |
|  | C2D18 | 48 | 26.5 | 37.4 | 33 | 30.2 | 70.6 | 23.9 | 40.8 | NS | 29.1 | 30.1 | 20.8 | 9.67 | 32.9 | 22.6 | 21.8 | 52 |
|  | C3D1-B | 52 | 16 | 25 | 27 | 21 | 34.4 | 16.7 | 26.3 | NS | 19.8 | 20.7 | 13.5 | 5.12 | 22.7 | 18.4 | 15.8 | 31.3 |

*, BLQ represented the absence of a plasma sample.

**Table S3.** Information of the 48 significantly changed metabolites between SH1 and C1D14.

| **Groups** | **Compound name** | **Category** | **HMDB ID.** | **Formula** | **P-value** |
| --- | --- | --- | --- | --- | --- |
| SH1 *VS* C1D14 | L-tyrosine | Amino acids and derivatives | HMDB0000158 | C9H11NO3 | 5.95E-09 |
| SH1 *VS* C1D14 | cortisol | Steroids | HMDB0000063 | C21H30O5 | 9.11E-07 |
| SH1 *VS* C1D14 | succinate | Organic acids and derivatives | HMDB0000254 | C4H6O4 | 1.64E-05 |
| SH1 *VS* C1D14 | pregnenolone | Steroids | HMDB0000253 | C21H32O2 | 1.98E-05 |
| SH1 *VS* C1D14 | hydroxyphenylpyruvate | Amino acids and derivatives | HMDB0011663 | C9H8O4 | 4.54E-05 |
| SH1 *VS* C1D14 | L-glutamine | Amino acids and derivatives | HMDB0000641 | C5H10N2O3 | 7.33E-05 |
| SH1 *VS* C1D14 | α-ketoglutarate | Organic acids and derivatives | HMDB0000208 | C5H6O5 | 2.33E-04 |
| SH1 *VS* C1D14 | L-alanine | Amino acids and derivatives | HMDB0000161 | C3H7NO2 | 6.93E-04 |
| SH1 *VS* C1D14 | L-arginine | Amino acids and derivatives | HMDB0000517 | C6H14N4O2 | 9.49E-04 |
| SH1 *VS* C1D14 | xanthosine | Nucleotides and derivatives | HMDB0000299 | C10H12N4O6 | 2.20E-03 |
| SH1 *VS* C1D14 | isobutyryl-L-carnitine | Acyl carnitines | HMDB0000736 | C11H21NO4 | 2.39E-03 |
| SH1 *VS* C1D14 | androstenedione | Steroids | HMDB0000053 | C19H26O2 | 2.61E-03 |
| SH1 *VS* C1D14 | 3, 5-tetradecadiencarnitine | Acyl carnitines | HMDB0013331 | C21H37NO4 | 5.51E-03 |
| SH1 *VS* C1D14 | octenoylcarnitine | Acyl carnitines | HMDB0013324 | C15H27NO4 | 5.66E-03 |
| SH1 *VS* C1D14 | decadienoylcarnitine | Acyl carnitines | HMDB0013325 | C17H29NO4 | 5.87E-03 |
| SH1 *VS* C1D14 | 5, 8-tetradecadiencarnitine | Acyl carnitines | - | C21H37NO4 | 6.28E-03 |
| SH1 *VS* C1D14 | 9-decenoylcarnitine | Acyl carnitines | HMDB0013205 | C17H31NO4 | 6.43E-03 |
| SH1 *VS* C1D14 | L-threonine | Amino acids and derivatives | HMDB0000167 | C4H9NO3 | 6.98E-03 |
| SH1 *VS* C1D14 | decanoyl-carnitine | Acyl carnitines | HMDB0000651 | C17H33NO4 | 8.11E-03 |
| SH1 *VS* C1D14 | 11-deoxycortisol | Steroids | HMDB0000015 | C21H30O4 | 8.43E-03 |
| SH1 *VS* C1D14 | L-lysine | Amino acids and derivatives | HMDB0000182 | C6H14N2O2 | 8.50E-03 |
| SH1 *VS* C1D14 | dodecanoylcarnitine | Acyl carnitines | HMDB0002250 | C19H37NO4 | 9.15E-03 |
| SH1 *VS* C1D14 | propanoyl-carnitine | Acyl carnitines | HMDB0062514 | C10H19NO4 | 1.16E-02 |
| SH1 *VS* C1D14 | 4-decenoylcarnitine | Acyl carnitines | - | C17H31NO4 | 1.18E-02 |
| SH1 *VS* C1D14 | 5-tetradecenoylcarnitine | Acyl carnitines | HMDB0002014 | C21H39NO4 | 1.22E-02 |
| SH1 *VS* C1D14 | L-isoleucine | Amino acids and derivatives | HMDB0000172 | C6H13NO2 | 1.27E-02 |
| SH1 *VS* C1D14 | L-leucine | Amino acids and derivatives | HMDB0000687 | C6H13NO2 | 1.27E-02 |
| SH1 *VS* C1D14 | L-kynurenine | Amino acids and derivatives | HMDB0000684 | C10H12N2O3 | 1.41E-02 |
| SH1 *VS* C1D14 | alpha-linolenyl carnitine | Acyl carnitines | HMDB0006319 | C23H39NO4 | 1.56E-02 |
| SH1 *VS* C1D14 | octanoyl-carnitine | Acyl carnitines | HMDB0000791 | C15H29NO4 | 1.61E-02 |
| SH1 *VS* C1D14 | xanthine | Nucleotides and derivatives | HMDB0000292 | C5H4N4O2 | 1.64E-02 |
| SH1 *VS* C1D14 | 4,8-dimethylnonanoyl carnitine | Acyl carnitines | HMDB0006202 | C18H35NO4 | 1.77E-02 |
| SH1 *VS* C1D14 | deoxyadenosine triphosphate | Nucleotides and derivatives | HMDB0001532 | C10H16N5O12P3 | 1.79E-02 |
| SH1 *VS* C1D14 | butanoyl-carnitine | Acyl carnitines | HMDB0002013 | C11H21NO4 | 1.79E-02 |
| SH1 *VS* C1D14 | dehydroepiandrosterone | Steroids | HMDB0000077 | C19H28O2 | 1.81E-02 |
| SH1 *VS* C1D14 | fumarate | Organic acids and derivatives | HMDB0000134 | C4H4O4 | 1.84E-02 |
| SH1 *VS* C1D14 | corticosterone | Steroids | HMDB0001547 | C21H30O4 | 2.09E-02 |
| SH1 *VS* C1D14 | undecanoylcarnitine | Acyl carnitines | HMDB0013321 | C18H35NO4 | 2.15E-02 |
| SH1 *VS* C1D14 | L-phenylalanine | Amino acids and derivatives | HMDB0000159 | C9H11NO2 | 2.23E-02 |
| SH1 *VS* C1D14 | trimethylamine-N-oxide | Bioamines and derivatives | HMDB0000925 | C3H9NO | 2.41E-02 |
| SH1 *VS* C1D14 | D-gluconic acid | Sugar and derivatives | HMDB0000625 | C6H12O7 | 2.93E-02 |
| SH1 *VS* C1D14 | L-valine | Amino acids and derivatives | HMDB0000883 | C5H11NO2 | 3.19E-02 |
| SH1 *VS* C1D14 | hexadecadienoylcarnitine | Acyl carnitines | HMDB0013334 | C23H41NO4 | 3.19E-02 |
| SH1 *VS* C1D14 | L-tryptophan | Amino acids and derivatives | HMDB0000929 | C11H12N2O2 | 3.43E-02 |
| SH1 *VS* C1D14 | cyclic AMP | Nucleotides and derivatives | HMDB0000058 | C10H12N5O6P | 3.51E-02 |
| SH1 *VS* C1D14 | docosadienoic acid | Fatty acids | HMDB0062219 | C22H40O2 | 3.69E-02 |
| SH1 *VS* C1D14 | inosine | Nucleotides and derivatives | HMDB0000195 | C10H12N4O5 | 3.99E-02 |
| SH1 *VS* C1D14 | L-proline | Amino acids and derivatives | HMDB0000162 | C5H9NO2 | 4.43E-02 |

**Table S4.** Obviously disturbed metabolic pathways owing to the treatment of anlotinib.

| **SH1 *VS* C1D14** | | |  | **SH1 *VS* C2D14** | | |
| --- | --- | --- | --- | --- | --- | --- |
| **Pathway Name** | **P-value** | **Impact** |  | **Pathway Name** | **P-value** | **Impact** |
| Aminoacyl-tRNA biosynthesis | 1.89E-11 | 0.0563 |  | Aminoacyl-tRNA biosynthesis | 2.06E-09 | 0.0563 |
| Alanine, aspartate and glutamate metabolism | 7.65E-06 | 0.2669 |  | Alanine, aspartate and glutamate metabolism | 5.63E-07 | 0.3124 |
| Phenylalanine, tyrosine and tryptophan biosynthesis | 2.82E-04 | 0.0080 |  | Purine metabolism | 0.0015 | 0.0460 |
| Valine, leucine and isoleucine biosynthesis | 2.82E-04 | 0.0398 |  | Nitrogen metabolism | 0.0019 | 0.0076 |
| Purine metabolism | 7.48E-04 | 0.0460 |  | Citrate cycle (TCA cycle) | 0.0025 | 0.1171 |
| Steroid hormone biosynthesis | 0.0011 | 0.2143 |  | Phenylalanine metabolism | 0.0033 | 0.1191 |
| Nitrogen metabolism | 0.0012 | 0.0000 |  | Arginine and proline metabolism | 0.0040 | 0.2486 |
| Citrate cycle (TCA cycle) | 0.0017 | 0.1171 |  | Phenylalanine, tyrosine and tryptophan biosynthesis | 0.0060 | 0.0080 |
| Phenylalanine metabolism | 0.0020 | 0.1191 |  | Valine, leucine and isoleucine biosynthesis | 0.0060 | 0.0265 |
| D-Glutamine and D-glutamate metabolism | 0.0077 | 0.0267 |  | D-Glutamine and D-glutamate metabolism | 0.0098 | 0.0267 |
| Valine, leucine and isoleucine degradation | 0.0127 | 0.0223 |  | Steroid hormone biosynthesis | 0.0115 | 0.1822 |
| Butanoate metabolism | 0.0127 | 0.0355 |  | Butanoate metabolism | 0.0178 | 0.0355 |
| Tyrosine metabolism | 0.0135 | 0.0894 |  | Tyrosine metabolism | 0.0208 | 0.0894 |
| Arginine and proline metabolism | 0.0141 | 0.2286 |  | Caffeine metabolism | 0.0344 | 0.0305 |
| Caffeine metabolism | 0.0272 | 0.0305 |  |  |  |  |

**Table S5.** Information of the 52 significantly changed metabolites between SH1 and C2D14.

| **Groups** | **Compound name** | **Category** | **HMDB ID.** | **Formula** | **P-value** |
| --- | --- | --- | --- | --- | --- |
| SH1 *VS* C2D14 | L-tyrosine | Amino acids and derivatives | HMDB0000158 | C9H11NO3 | 1.86E-09 |
| SH1 *VS* C2D14 | succinate | Organic acids and derivatives | HMDB0000254 | C4H6O4 | 9.33E-07 |
| SH1 *VS* C2D14 | hydroxyphenylpyruvate | Amino acids and derivatives | HMDB0011663 | C9H8O4 | 1.83E-05 |
| SH1 *VS* C2D14 | cortisol | Steroids | HMDB0000063 | C21H30O5 | 2.43E-05 |
| SH1 *VS* C2D14 | pregnenolone | Steroids | HMDB0000253 | C21H32O2 | 6.48E-05 |
| SH1 *VS* C2D14 | L-glutamine | Amino acids and derivatives | HMDB0000641 | C5H10N2O3 | 8.55E-05 |
| SH1 *VS* C2D14 | α-ketoglutarate | Organic acids and derivatives | HMDB0000208 | C5H6O5 | 9.88E-05 |
| SH1 *VS* C2D14 | L-alanine | Amino acids and derivatives | HMDB0000161 | C3H7NO2 | 3.86E-04 |
| SH1 *VS* C2D14 | decadienoylcarnitine | Acyl carnitines | HMDB0013325 | C17H29NO4 | 4.24E-04 |
| SH1 *VS* C2D14 | xanthine | Nucleotides and derivatives | HMDB0000292 | C5H4N4O2 | 5.07E-04 |
| SH1 *VS* C2D14 | cyclic AMP | Nucleotides and derivatives | HMDB0000058 | C10H12N5O6P | 5.58E-04 |
| SH1 *VS* C2D14 | butanoyl-carnitine | Acyl carnitines | HMDB0002013 | C11H21NO4 | 1.36E-03 |
| SH1 *VS* C2D14 | 4,8-dimethylnonanoyl carnitine | Acyl carnitines | HMDB0006202 | C18H35NO4 | 1.62E-03 |
| SH1 *VS* C2D14 | inosine | Nucleotides and derivatives | HMDB0000195 | C10H12N4O5 | 1.89E-03 |
| SH1 *VS* C2D14 | 9-decenoylcarnitine | Acyl carnitines | HMDB0013205 | C17H31NO4 | 2.30E-03 |
| SH1 *VS* C2D14 | L-proline | Amino acids and derivatives | HMDB0000162 | C5H9NO2 | 2.36E-03 |
| SH1 *VS* C2D14 | L-isoleucine | Amino acids and derivatives | HMDB0000172 | C6H13NO2 | 2.48E-03 |
| SH1 *VS* C2D14 | L-leucine | Amino acids and derivatives | HMDB0000687 | C6H13NO2 | 2.48E-03 |
| SH1 *VS* C2D14 | dodecanoylcarnitine | Acyl carnitines | HMDB0002250 | C19H37NO4 | 3.20E-03 |
| SH1 *VS* C2D14 | decanoyl-carnitine | Acyl carnitines | HMDB0000651 | C17H33NO4 | 4.25E-03 |
| SH1 *VS* C2D14 | 4-decenoylcarnitine | Acyl carnitines | - | C17H31NO4 | 4.93E-03 |
| SH1 *VS* C2D14 | L-lysine | Amino acids and derivatives | HMDB0000182 | C6H14N2O2 | 5.55E-03 |
| SH1 *VS* C2D14 | 11-deoxycortisol | Steroids | HMDB0000015 | C21H30O4 | 5.87E-03 |
| SH1 *VS* C2D14 | DL-pyroglutamic acid | Amino acids and derivatives | HMDB0000267 | C5H7NO3 | 5.95E-03 |
| SH1 *VS* C2D14 | L-arginine | Amino acids and derivatives | HMDB0000517 | C6H14N4O2 | 5.98E-03 |
| SH1 *VS* C2D14 | 5, 8-tetradecadiencarnitine | Acyl carnitines | - | C21H37NO4 | 6.11E-03 |
| SH1 *VS* C2D14 | deoxyadenosine triphosphate | Nucleotides and derivatives | HMDB0001532 | C10H16N5O12P3 | 6.56E-03 |
| SH1 *VS* C2D14 | 3, 5-tetradecadiencarnitine | Acyl carnitines | HMDB0013331 | C21H37NO4 | 7.42E-03 |
| SH1 *VS* C2D14 | octenoylcarnitine | Acyl carnitines | HMDB0013324 | C15H27NO4 | 9.73E-03 |
| SH1 *VS* C2D14 | octanoyl-carnitine | Acyl carnitines | HMDB0000791 | C15H29NO4 | 1.01E-02 |
| SH1 *VS* C2D14 | carnitine | Amino acids and derivatives | HMDB0000062 | C7H15NO3 | 1.04E-02 |
| SH1 *VS* C2D14 | xanthosine | Nucleotides and derivatives | HMDB0000299 | C10H12N4O6 | 1.16E-02 |
| SH1 *VS* C2D14 | androstenedione | Steroids | HMDB0000053 | C19H26O2 | 1.20E-02 |
| SH1 *VS* C2D14 | L-threonine | Amino acids and derivatives | HMDB0000167 | C4H9NO3 | 1.24E-02 |
| SH1 *VS* C2D14 | valeroyl-carnitine | Acyl carnitines | HMDB0013128 | C12H23NO4 | 1.31E-02 |
| SH1 *VS* C2D14 | undecanoylcarnitine | Acyl carnitines | HMDB0013321 | C18H35NO4 | 1.47E-02 |
| SH1 *VS* C2D14 | N,N-dimethylglycine | Amino acids and derivatives | HMDB0000092 | C4H9NO2 | 2.31E-02 |
| SH1 *VS* C2D14 | glycoursodeoxycholic acid | Biel acid | HMDB0000708 | C26H43NO5 | 2.34E-02 |
| SH1 *VS* C2D14 | uracil | Nucleotides and derivatives | HMDB0000300 | C4H4N2O2 | 2.56E-02 |
| SH1 *VS* C2D14 | fumarate | Organic acids and derivatives | HMDB0000134 | C4H4O4 | 2.57E-02 |
| SH1 *VS* C2D14 | fumarycarnitine | Acyl carnitines | HMDB0013134 | C11H17NO6 | 2.69E-02 |
| SH1 *VS* C2D14 | propanoyl-carnitine | Acyl carnitines | HMDB0062514 | C10H19NO4 | 2.81E-02 |
| SH1 *VS* C2D14 | tetradecanoylcarnitine | Acyl carnitines | HMDB0005066 | C21H41NO4 | 3.13E-02 |
| SH1 *VS* C2D14 | uridine | Nucleotides and derivatives | HMDB0000296 | C9H12N2O6 | 3.47E-02 |
| SH1 *VS* C2D14 | alpha-linolenyl carnitine | Acyl carnitines | HMDB0006319 | C23H39NO4 | 3.81E-02 |
| SH1 *VS* C2D14 | N-acetyl-D-glucosamine | Sugar and derivatives | HMDB0000215 | C8H15NO6 | 3.84E-02 |
| SH1 *VS* C2D14 | L-phenylalanine | Amino acids and derivatives | HMDB0000159 | C9H11NO2 | 3.97E-02 |
| SH1 *VS* C2D14 | citrulline | Amino acids and derivatives | HMDB0000904 | C6H13N3O3 | 4.08E-02 |
| SH1 *VS* C2D14 | L-lactic acid | Organic acids and derivatives | HMDB0000190 | C3H6O3 | 4.58E-02 |
| SH1 *VS* C2D14 | glycerol | Sugar and derivatives | HMDB0000131 | C3H8O3 | 4.69E-02 |
| SH1 *VS* C2D14 | L-asparagine | Amino acids and derivatives | HMDB0000168 | C4H8N2O3 | 4.77E-02 |
| SH1 *VS* C2D14 | corticosterone | Steroids | HMDB0001547 | C21H30O4 | 4.79E-02 |

**Table S6.** Information of 38 significantly changed metabolites after anlotinib treatment.

| **Compound name** | **Category** | **HMDB ID.** | **Formula** | **P-value** | |
| --- | --- | --- | --- | --- | --- |
|  |  |  |  | **SH1-C1D14** | **SH1-C2D14** |
| L-tyrosine | Amino acids and derivatives | HMDB0000158 | C9H11NO3 | 5.95E-09 | 1.86E-09 |
| cortisol | Steroids | HMDB0000063 | C21H30O5 | 9.11E-07 | 2.43E-05 |
| succinate | Organic acids and derivatives | HMDB0000254 | C4H6O4 | 1.64E-05 | 9.33E-07 |
| pregnenolone | Steroids | HMDB0000253 | C21H32O2 | 1.98E-05 | 6.48E-05 |
| hydroxyphenylpyruvate | Amino acids and derivatives | HMDB0011663 | C9H8O4 | 4.54E-05 | 1.83E-05 |
| L-glutamine | Amino acids and derivatives | HMDB0000641 | C5H10N2O3 | 7.33E-05 | 8.55E-05 |
| α-ketoglutarate | Organic acids and derivatives | HMDB0000208 | C5H6O5 | 2.33E-04 | 9.88E-05 |
| L-alanine | Amino acids and derivatives | HMDB0000161 | C3H7NO2 | 6.93E-04 | 3.86E-04 |
| L-arginine | Amino acids and derivatives | HMDB0000517 | C6H14N4O2 | 9.49E-04 | 5.98E-03 |
| xanthosine | Nucleotides and derivatives | HMDB0000299 | C10H12N4O6 | 2.20E-03 | 1.16E-02 |
| androstenedione | Steroids | HMDB0000053 | C19H26O2 | 2.61E-03 | 1.20E-02 |
| 3, 5-tetradecadiencarnitine | Acyl carnitines | HMDB0013331 | C21H37NO4 | 5.51E-03 | 7.42E-03 |
| octenoylcarnitine | Acyl carnitines | HMDB0013324 | C15H27NO4 | 5.66E-03 | 9.73E-03 |
| decadienoylcarnitine | Acyl carnitines | HMDB0013325 | C17H29NO4 | 5.87E-03 | 4.24E-04 |
| 5, 8-tetradecadiencarnitine | Acyl carnitines | - | C21H37NO4 | 6.28E-03 | 6.11E-03 |
| 9-decenoylcarnitine | Acyl carnitines | HMDB0013205 | C17H31NO4 | 6.43E-03 | 2.30E-03 |
| L-threonine | Amino acids and derivatives | HMDB0000167 | C4H9NO3 | 6.98E-03 | 1.24E-02 |
| decanoyl-carnitine | Acyl carnitines | HMDB0000651 | C17H33NO4 | 8.11E-03 | 4.25E-03 |
| 11-deoxycortisol | Steroids | HMDB0000015 | C21H30O4 | 8.43E-03 | 5.87E-03 |
| L-lysine | Amino acids and derivatives | HMDB0000182 | C6H14N2O2 | 8.50E-03 | 5.55E-03 |
| dodecanoylcarnitine | Acyl carnitines | HMDB0002250 | C19H37NO4 | 9.15E-03 | 3.20E-03 |
| propanoyl-carnitine | Acyl carnitines | HMDB0062514 | C10H19NO4 | 1.16E-02 | 2.81E-02 |
| 4-decenoylcarnitine | Acyl carnitines | - | C17H31NO4 | 1.18E-02 | 4.93E-03 |
| L-isoleucine | Amino acids and derivatives | HMDB0000172 | C6H13NO2 | 1.27E-02 | 2.48E-03 |
| L-leucine | Amino acids and derivatives | HMDB0000687 | C6H13NO2 | 1.27E-02 | 2.48E-03 |
| alpha-linolenyl carnitine | Acyl carnitines | HMDB0006319 | C23H39NO4 | 1.56E-02 | 3.81E-02 |
| octanoyl-carnitine | Acyl carnitines | HMDB0000791 | C15H29NO4 | 1.61E-02 | 1.01E-02 |
| xanthine | Nucleotides and derivatives | HMDB0000292 | C5H4N4O2 | 1.64E-02 | 5.07E-04 |
| 4,8-dimethylnonanoyl carnitine | Acyl carnitines | HMDB0006202 | C18H35NO4 | 1.77E-02 | 1.62E-03 |
| butanoyl-carnitine | Acyl carnitines | HMDB0002013 | C11H21NO4 | 1.79E-02 | 1.36E-03 |
| dATP | Nucleotides and derivatives | HMDB0001532 | C10H16N5O12P3 | 1.79E-02 | 6.56E-03 |
| fumarate | Organic acids and derivatives | HMDB0000134 | C4H4O4 | 1.84E-02 | 2.57E-02 |
| corticosterone | Steroids | HMDB0001547 | C21H30O4 | 2.09E-02 | 4.79E-02 |
| undecanoylcarnitine | Acyl carnitines | HMDB0013321 | C18H35NO4 | 2.15E-02 | 1.47E-02 |
| L-phenylalanine | Amino acids and derivatives | HMDB0000159 | C9H11NO2 | 2.23E-02 | 3.97E-02 |
| 3’, 5’-cyclic AMP | Nucleotides and derivatives | HMDB0000058 | C10H12N5O6P | 3.51E-02 | 5.58E-04 |
| inosine | Nucleotides and derivatives | HMDB0000195 | C10H12N4O5 | 3.99E-02 | 1.89E-03 |
| L-proline | Amino acids and derivatives | HMDB0000162 | C5H9NO2 | 4.43E-02 | 2.36E-03 |

**Table S7.** Tumor volume during different treatment cycles of the 13 subjects involved in anlotinib efficacy study.

| **Group** | **Subject NO.** | **Cycles of the 2-week on/1-week off protocol** | | | | | **Best curative effect***  **（%）** |
| --- | --- | --- | --- | --- | --- | --- | --- |
|  |  | **Baseline** | **The 2^nd^** | **The 4^th^** | **The 6^th^** | **The 8^th^** |  |
| Good efficacy group | 005 | 141 | 125 | 104 | 99 |  | -29.7 |
|  | 001 | 38 | 29 | 29 | 28 | 27 | -28.9 |
|  | 004 | 66 | 55 | 50 | 50 |  | -24.2 |
|  | 003 | 224 | 208 | 192 | 211 |  | -14.2 |
|  | 010 | 89 | 88 | 79 |  |  | -11.2 |
|  | 011 | 140 | 130 | 126 |  |  | -10 |
| Poor efficacy group | 016 | 109 | 99 |  |  |  | -9.1 |
|  | 006 | 15 | 15 | 14 | 14 |  | -6.6 |
|  | 009 | 41 | 40 | 39 |  |  | -4.8 |
|  | 002 | 26 | 26 | 25 | 26 | 28 | -3.8 |
|  | 012 | 117 | 110 | 112 |  |  | -0.59 |
|  | 007 | 26 | 27 | 28 | 30 |  | 3.8 |
|  | 014 | 108 | 145 |  |  |  | 34.2 |

*, The best curative effect is an important evaluation index of objective remission rate of tumor and it can be calculated by the following formula: (minimum value of tumor volume-baseline value of tumor volume)×100% / baseline value of tumor volume.

**Table S8.** Detail information of potential biomarkers related to the efficacy of anlotinib.

| **Name** | **HMDB ID.** | **Formula** | **VIP** | **P-value** | **AUC** | **Fold change** |
| --- | --- | --- | --- | --- | --- | --- |
| Glycodeoxycholic acid | HMDB0000631 | C26H43NO5 | 2.29 | 1.04E-16 | 0.85 | 0.25 |
| Glycocholic acid | HMDB0000138 | C26H43NO6 | 2.25 | 6.70E-11 | 0.83 | 0.18 |
| Glycochenodeoxycholic acid | HMDB0000637 | C26H43NO5 | 1.92 | 1.94E-12 | 0.77 | 0.26 |
| Ornithine | HMDB0000214 | C5H12N2O2 | 1.87 | 1.30E-17 | 0.79 | 0.65 |
| Deoxycholic acid | HMDB0000626 | C24H40O4 | 1.86 | 1.43E-11 | 0.76 | 0.49 |
| Taurochenodeoxycholic acid | HMDB0000951 | C26H45NO6S | 1.79 | 8.56E-10 | 0.75 | 0.36 |
| Betaine | HMDB0000043 | C5H11NO2 | 1.72 | 3.54E-16 | 0.76 | 0.73 |
| Hypoxanthine | HMDB0000157 | C5H4N4O | 1.71 | 6.74E-10 | 0.75 | 1.41 |
| Choline | HMDB0000097 | C5H14NO | 1.70 | 7.01E-16 | 0.75 | 0.81 |
| L-Phenylalanine | HMDB0000159 | C9H11NO2 | 1.69 | 7.91E-15 | 0.74 | 0.88 |
| L-Threonine | HMDB0000167 | C4H9NO3 | 1.62 | 1.70E-14 | 0.74 | 0.83 |
| Taurine | HMDB0000251 | C2H7NO3S | 1.57 | 6.85E-11 | 0.71 | 1.22 |

**Figure S1.** Anlotinib blood concentrations of each subjects.

**Figure S2.** Concentration trends of L-tyrosine in 16 subjects based on longitudinal pharmacometabonomics data. The concentration of L-tyrosine on time point SH1 was defined as 1, and the concentrations on the other time points were normalized by the concentration on SH1.

**Figure S3.** Histograms and ROC curves of NG,NG-dimethylarginine based on concentrations from different time points. (A) Histogram and ROC curve based on NG,NG-dimethylarginine concentrations from SH1 and SH2. (B) Histogram and ROC curve based on NG,NG-dimethylarginine concentrations from SH1, SH2 and SH4. (C) Histogram and ROC curve based on NG,NG-dimethylarginine concentrations from SH1, SH2, SH4, SH8, SH11 and SH24. (D) Histogram and ROC curve based on NG,NG-dimethylarginine concentrations from SH1, SH2, SH4, SH8, SH11, SH24, SH48, SH72, SH120, SH168 and SH240.
